# Biological properties and conformational studies of amphiphilic Pd(II) and Ni(II) complexes bearing functionalized aroylaminocarbo-*N*-thioylpyrrolinate units

**DOI:** 10.3762/bjoc.17.192

**Published:** 2021-12-02

**Authors:** Samet Poyraz, Samet Belveren, Sabriye Aydınoğlu, Mahmut Ulger, Abel de Cózar, Maria de Gracia Retamosa, Jose M Sansano, H Ali Döndaş

**Affiliations:** 1 Department of Chemistry, Faculty of Pharmacy, Mersin University, Yenisehir, 33169 Mersin, Turkey; 2 Department of Basic Pharmaceutical Sciences, Faculty of Pharmacy, Çukurova University, Balcalı 01330, Adana, Turkey; 3 Department of Pharmaceutical Microbiology, Faculty of Pharmacy, Mersin University, Yenisehir, 33169 Mersin, Turkey; 4 Departamento de Química Orgánica I, Facultad de Química. Universidad del País Vasco/Euskal Herriko Unibertsitatea UPV/EHU, Centro de Innovación en Química Avanzada (ORFEO-CINQA) and Donostia International Physics Center (DIPC), P. K. 1072, E-20018 San Sebastián, Spain; 5 IKERBASQUE, Basque Foundation of Science, Plaza Euskadi 5, 48009, Bilbao, Spain; 6 University of Alicante, Department of Organic Chemistry, Centro de Innovación en Química Avanzada (ORFEO-CINQA) and Instituto de Síntesis Orgánica (ISO), PO Box 99, 03080 Alicante, Spain

**Keywords:** antituberculosis, bidentate ligands, DFT, nickel, palladium

## Abstract

A series of novel palladium(II) and nickel(II) complexes of multifunctionalized aroylaminocarbo-*N*-thioylpyrrolinates were synthesized and characterized by analytical and spectroscopic techniques. The biological properties of the freshly prepared compounds were screened against *S. aureus*, *B. subtilis*, *A. hydrophila*, *E. coli*, and *A. baumannii* bacteria and antituberculosis activity against *M. tuberculosis* H37Rv strains. Also, the antifungal activity was studied against *C. albicans*, *C. tropicalis*, and *C. glabrata* standard strains. A deep conformational survey was monitored using DFT calculations with the aim to explain the importance of the final conformation in the biological experimental results.

## Introduction

In recent years, metal complexes with biological activity are of paramount relevance in medicine as valuable alternatives for the classical pharmaceuticals based on organic compound scaffolds. In this line, metal complexes incorporating an amphiphilic character (also called metallosurfactants) exhibit very interesting properties, but their biological and medicinal applications have not been fully developed yet [1–2]. One of the most promising areas of interest in medicine is oncology [3–5] and infectious diseases [6–8] (or both) [9]. The modulation of the hydrophobicity domain, the influence of the strength of the ligand and the metal cation sphere (together with small molecules coordinated in its outer sphere) are crucial points to study the structure–activity relationship (SAR) onto a fixed biological target [10–12]. In particular, *N*-benzoylthiourea derivatives are versatile ligands that could coordinate several metal centers with the aid of sulfur, nitrogen or oxygen donor atoms that allow multiple bindings [13]. Moreover, compounds bearing a –C(O)NHC(S)– moiety and their metal complexes have assorted biological and pharmacological properties such as anti(myco)bacterial, antitumoral, antimalarial, antifungal, or antiviral activities [14].

In this study, as a continuation of our work related to organometallic compounds with very low/modest amphiphilic character [15–17], we propose the incorporation of a 3-indolylmethyl group in the ligand and compare the bioactivity results of its corresponding nickel(II) [18] and palladium(II) [19–20] complexes with the analogous tests obtained in the case of having a benzylic substituent at the same position. Also, DFT calculations are run in order to study the conformational analysis of the synthetized complexes.

## Results and Discussion

### Synthesis of **L1-M**, **L2-M**, and **L3-M**

**Scheme 1:**
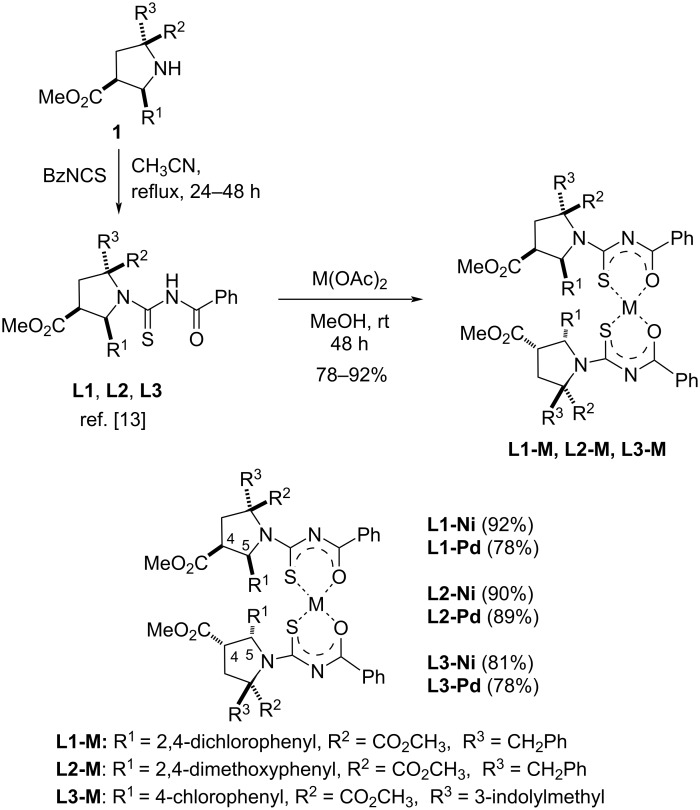
Synthetic route for the preparation of **L1-M**, **L2-M** and **L3-M** complexes.

According to our experience, concerning the most bioactive structural arrangement, the ligands **L1**, **L2**, and **L3** were selected for this study (Scheme 1). Using the known methodology developed by our group, the starting compounds *endo-*prolinates **1**, generated by 1,3-dipolar cycloaddition [16,21–22], were submitted to the reaction with benzoyl isothiocyanate in refluxing acetonitrile to obtain compounds **L1**, **L2** and **L3** in good yields [16,21]. Due to the very low biological activity of these ligands by themselves, the chelation with nickel(II) and palladium(II) was successfully performed using the corresponding acetate salt in methanol at rt for 48 h, affording *cis-***L1-M**, **L2-M**, and **L3-M** in yields depicted in Scheme 1. The relative configuration was unambiguously determined by comparison of their corresponding ^1^H NMR with the reported similar ^1^H NMR with other metals, and the *cis-*configuration determined according to X-ray diffraction patterns observed in precedent works [15–16]. Although a structural motif of the metallosurfactants is having a long alkyl chain (hydrophobic part), in these cases, **L1**–**L3** possessed four hydrophobic domains, which are responsible of the formation of micellar aggregates in water, they were very difficult to recrystallize. These two experimental facts supported the employment of a metallosurfactant definition to the complexes described here.

Rotamers of the ligands **L1**–**L3** observed in the ^1^H NMR spectra disappeared in large extension after complexation. NH (amide) protons of the ligands located at 8.30–7.65 ppm were not detected in the metallic structure spectra. Signals in the ranges of 5.84–5.64 and 4.40–4.17 ppm, corresponding to H5 and H6 protons, respectively, were shifted to 5.44 (H5) and 4.19 (H6) ppm for **L1-Ni**, and to 5.56–5.48 and 4.29–4.23 for the **L1-Pd** complex. In addition, the ^1^H and ^13^C NMR spectra of nickel(II) complexes (**L1-Ni**, **L2-Ni**, and **L3-Ni**) show clear and simple signals of protons and carbons. However, the palladium complexes derived from ligands **L1** and **L2** exhibited major and minor sets of signals in both NMR experiments. This could be attributed to the distorted square planar geometry of the palladium complexes [23–26], compared to the square planar structure of the analogous nickel(II) chelates [15,27]. In fact, the sets of the signals were reduced upon warming the NMR probe.

Next, these freshly prepared organometallic compounds were screened for their antibacterial activity against a range of Gram-positive (*Staphylococcus aureus*, *Bacillus subtilis*) and Gram-negative (*Aeromonas hydrophila*, *Escherichia coli*, *Acinetobacter baumannii*) bacteria and antimycobacterial activity against *M. tuberculosis* H37Rv strains. Antifungal activity of the novel compounds was also evaluated against *Candida albicans*, *Candida tropicalis* and *Candida glabrata* strains.

### Antibacterial and antituberculosis (TB) activities

From the observed data (Table 1) it can be seen that the tested compounds showed moderate antibacterial activity, in the range of 15.62–250 μg/mL when compared to reference drugs. The **L3-Ni** complex possessing the indole ring is the most active compound among the other complexes against *Bacillus subtilis* with a value of 15.62 μg/mL. The activities of the samples were compared with the results obtained with ampicillin (0.9–125 μg/mL) as reference.

**Table 1:** Antibacterial and antituberculosis activity (μg/mL).

	*Staphylococcus aureus* (ATCC-25925)	*Escherichia coli* (ATCC-25923)	*Acinetobacter baumannii* (ATCC-02026)	*Bacillus subtilis* (ATCC-6633)	*Aeromonas hydrophila* (ATCC-95080)	*M. tuberculosis* H37Rv

**L1-Ni**	250	250	250	125	250	62.50
**L2-Ni**	250	250	250	250	250	62.50
**L3-Ni**	**250**	**250**	**250**	**15.62**	**250**	**3.90**
**L1-Pd**	250	250	250	250	250	62,50
**L2-Pd**	250	250	250	250	250	62.50
**L3-Pd**	250	250	250	250	250	62.50
ampicillin	31.25	15.62	125	0.9	31.25	
isoniazid						0,12 µg/mL
rifampicin						0.97 µg/mL

The tested complexes showed antituberculosis activity, in the range of 3.90–62,50 μg/mL (Table 1) when compared to reference drugs. It is interestingly important to notice that the **L3-Ni** complex bearing an indole ring in the pyrrolidine skeleton has the highest activity when tested against the *M. tuberculosis* H37Rv strain with 3.90 μg/mL. It seems that the presence of the indole ring has enhanced the activity of complex **L3-Ni** when comparing with other Ni complexes such as **L1-Ni** and **L2-Ni**.

### Antifungal activity

The screened complexes showed antifungal activity, in the range of 62.50–125 μg/mL (Table 2) when compared to reference drugs. the **L3-Ni** complex has the highest activity against *Candida glabrata.*

**Table 2:** Antifungal activity (μg/mL).

	*Candida albicans* ATCC 14053	*Candida tropicalis* ATCC 1969	*Candida glabrata* ATCC 15126

**L1-Ni**	125	125	125
**L2-Ni**	125	125	125
**L3-Ni**	**125**	**125**	**62.50**
**L1-Pd**	125	125	125
**L2-Pd**	125	125	125
**L3-Pd**	125	125	125
fluconazole	31.25	15.62	3.90

### DFT calculations of complexes

The survey of relationship between structure of the complex and activity (SAR) moved us to design DFT calculations. The difference of the conformations analyzed by the ^1^H NMR spectra of nickel and palladium complexes, presumably correlated with their activity. According to the previous activities of derivatives of this nature [15–16] the *cis-*complex was identified as the therapeutic arrow. We initially tried to determine the driving force causing of the selective formation of the *cis-* versus the *trans*-aggregate observed after X-ray diffraction analysis, but calculations did not give any clear interaction to confirm it. The polar solvent methanol used for the reaction and separation could favor this chemical arrangement.

**Figure 1:**
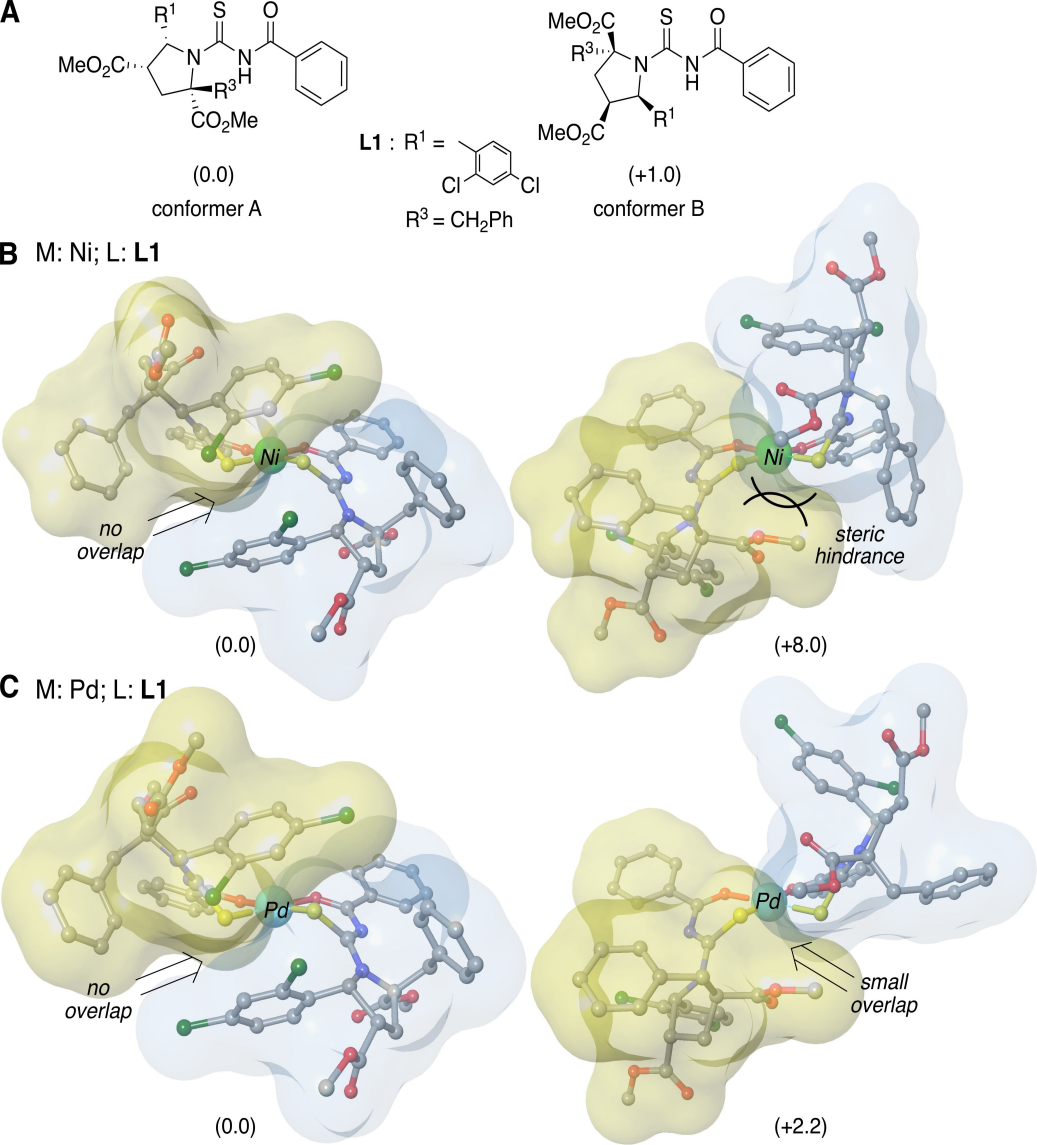
Main geometrical features and the relative energies (in kcal·mol^–1^) of (A) ligand **L1**, (B) nickel- and (C) palladium complexes. Blue and yellow surfaces represent the solvent accessible surface of ligands with a probe radius of 1.4 Å. Hydrogen atoms are omitted for clarity.

**Figure 2:**
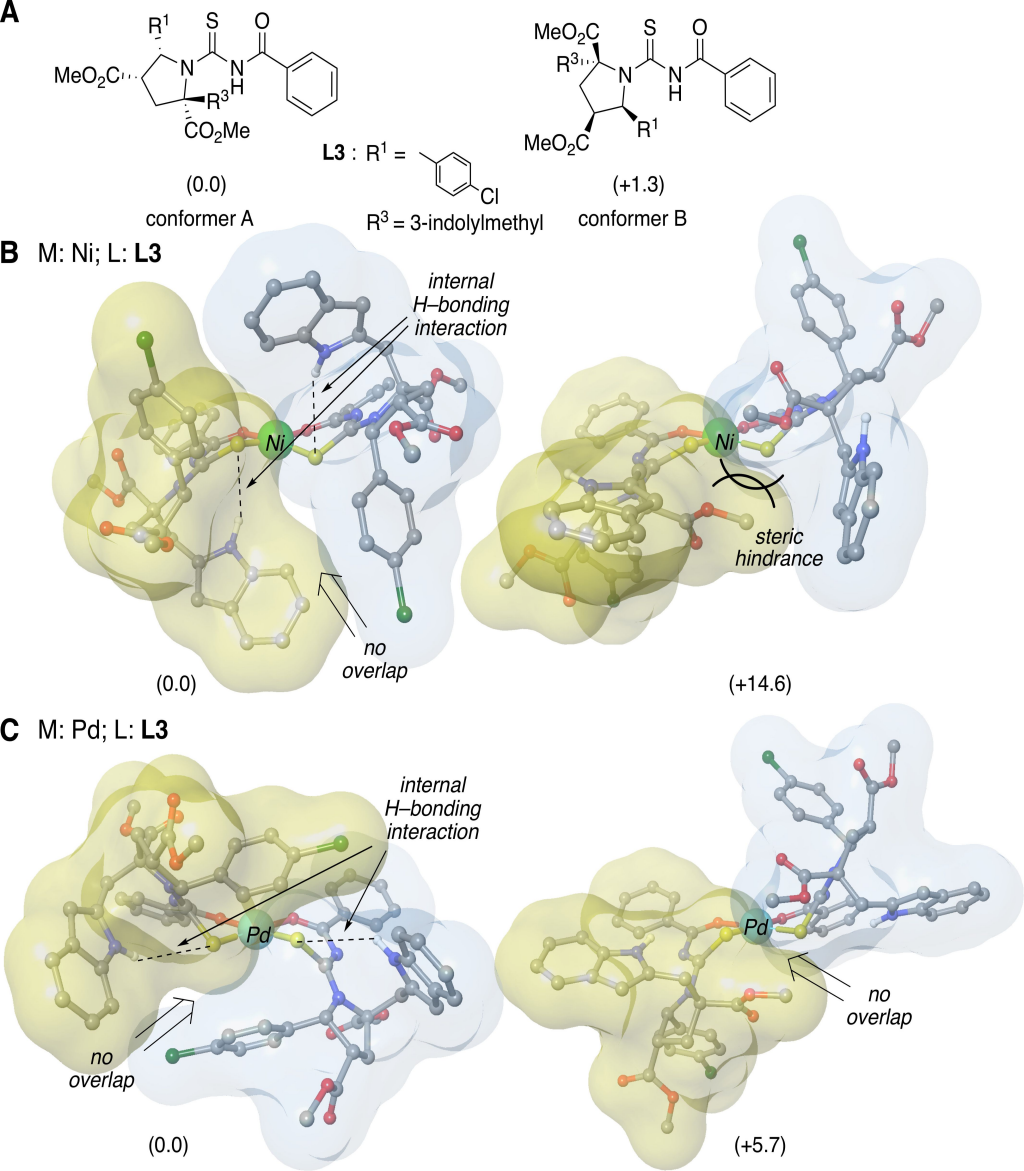
Main geometrical features and the relative energies (in kcal mol^–1^) of (A) ligand **L3**, (B) nickel- and (C) palladium complexes. Blue and yellow surfaces represent the solvent accessible surface of ligands with a probe radius of 1.4 Å. Hydrogen atoms not involved on hydrogen–bonding interactions are omitted for clarity.

Ligands **L1** and **L3** as well as nickel- and palladium-derived complexes were optimized at B3LYP-GD3BJ/TZVP and B3LYP-GD3BJ/TZVP &SDD level of theory, respectively. The presence of the benzyl and 3-indolylmethyl groups is crucial in their efficiency as drugs. In order to have a complete overview of the systems, different ligand rotamers were considered during the optimization process. Only one enantiomer was selected for running the calculations for simplicity. The obtained results are gathered in Figure 1 and Figure 2. These calculations show that for ligands **L1** and **L3**, conformer A and conformer B, resulting from a free rotation of the thioketone carbon–N-pyrrolidine bond, are energetically accessible, in agreement with the two set of signals observed by ^1^H NMR spectroscopy (energy differences between conformers lower than 1 kcal·mol^–1^ in both cases). However, this equilibrium is strongly affected by the coordination to nickel or palladium atoms. As far as the **L1-Ni** complex was concerned, this fluxional equilibrium disappeared due to metal coordination. In this case, complexes involving conformer B were highly energetic due to steric repulsion between the CO_2_Me substituents of the pyrrolidine ring. Thus, only one set of signals in NMR spectra (corresponding to conformer A) would be observed. For **L1-Pd** complexes, the aggregate included two energetically accessible rotamers at room temperature (energetic difference of +2.2 kcal·mol^−1^, Figure 1). Therefore, the duplicated set of signals in the ^1^H NMR was justified. We postulated that the longer metal–O and metal–S distances of the palladium atom, compared to nickel, placed the substituents of the pyrrolidine ring far enough to reduce the steric hindrance that destabilized nickel-conformer B complexes.

The results obtained for **L3**-**M** complexes showed that, independently of the metal atom considered, only one of the ligand conformations were energetically accessible (energetic difference of +14.6 and +5.7 kcal·mol^−1^, for nickel and palladium conformers, respectively, Figure 2). In these cases, the energetic difference between conformers A and B lies in the existence of intramolecular hydrogen-bonding interactions (Figure 2). Additional steric hindrance in conformer B, compared to conformer A (as found in **L1-Ni** complexes), raises the energy of the latter. Thus, in **L3-M** complexes, only one set of signals was expected in the NMR spectra.

Due to the high number of possible cellular targets known for antituberculosis drugs and all the different chemical structures (inhibition of cell wall synthesis, disruption of the plasma membrane, DNA-gyrase, etc.) the next work focused on determining the exact biological mechanism and docking studies could not be executed.

## Conclusion

The anti(myco)bacterial and antifungal activity of newly prepared organometallic compounds were investigated against some Gram-positive, Gram-negative bacteria, M. tuberculosis H37Rv strains and *C. albicans*, *C. tropicalis*, *C. glabrata* fungi. The screened novel complexes showed different degrees of activities in relation to the tested microorganisms together depending on the type of synthesized compounds. In general, the most active compound was the **L3-Ni** complex possessing the indole ring has a single conformation (detected by ^1^H NMR spectroscopy and confirmed by DFT calculations), which corresponded with a non-*meso* form being the responsible of this exclusive biological activity. Studies to determine the therapeutic target are underway.

## Experimental

### General

All commercially available reagents and solvents were used without further purification. Flash column chromatography was performed using silica gel 60 (230–400 mesh). Kieselgel columns were packed with silica gel GF254 (Merck 7730). Flash chromatography was carried out on handpacked columns of Merck silica gel 60 (0.040–0.063 mm). Melting points were determined on a Stuart SMP3 hot stage apparatus. The structurally most important peaks of the IR spectra (recorded using a Nicolet 510 P-FT) are listed and wave numbers are given in cm^−1^. Nuclear magnetic resonance spectra and decoupling experiments were determined at 250 MHz on a Q.E 300 instrument, at 300 MHz on a Bruker Avance AC-300 and at 500 MHz on a Bruker AM500 spectrometer as specified. Chemical shifts are given in parts per million (δ) downfield from tetramethylsilane as internal standard. Spectra were determined in CDCl_3_. The following abbreviations are used to describe peak patterns where appropriate: s = singlet, d = doublet, t = triplet, q = quartet, m = multiplet. All coupling constants (*J*) are given in Hz and chemical shifts in ppm. Low-resolution electron impact (EI) mass spectra were obtained at 70 eV using a Shimadzu QP-5000 by injection or DIP; fragment ions in *m*/*z* are given with relative intensities (%) in parentheses. High-resolution mass spectra (HRMS) were measured on an instrument using a quadrupole time-of-flight mass spectrometer (QTOF) and also through the electron impact mode (EI) at 70 eV using a Finnigan VG Platform or a Finnigan MAT 95S. VCD analysis was recorded in a Jasco FVS-6000. Microanalyses were measured in CHNS apparatus with a Micro TruSpec from LECO detection system.

### General procedure for ligands and complexes

**Synthesis of selected ligands (L1–L3**): The known pyrrolidines and aroylaminocarbo-*N*-thioylpyrrolidine compounds (**L1–L3**) were synthesized according to published procedures [15].

**Preparation of Ni(II) and Pd(II) complexes (L1-M, L2-M, L3-M):** A solution of metal acetate (0.1 mmol) in methanol (10 mL) was added dropwise to the solution of aroylaminocarbo-*N*-thioylpyrrolidine ligands (0.2 mmol) in methanol (25 mL) at room temperature. The resulting mixture was stirred for 48 h and the precipitated complexes were filtered and washed with methanol.

**{Dimethyl (2*RS*,4*SR*,5*RS*)-1-(benzoylcarbamothioyl)-2-benzyl-5-(2,4-dichlorophenyl)pyrrolidine-2,4-dicarboxylate}_2_Ni (L1-Ni):** Red solid, 113 mg, 92% yield; mp 321–323 °C (MeOH, decomp.); ^1^H NMR (400 MHz) δ 8.05 (d, *J* = 6.6 Hz, 4H), 7.92 (d, *J* = 8.2 Hz, 2H), 7.60–7.11 (m, 20H), 5.44 (d, *J* = 8.9 Hz, 2H-5), 4.19 (d, *J* = 13.8 Hz, 2H-6), 3.76 (s, 6H), 3.38 (d, *J* = 14.1 Hz, 2H), 3.20 (s, 6H), 3.00–2.81 (m, 2H), 2.35–2.15 (m, 4H); ^13^C NMR (100 MHz) δ 173.6 (2CS), 173.4 (2CO), 173.1 (2CO), 169.6 (2CO), 136.6 (2C), 136.1 (2C), 135.2 (2C), 134.1 (2C), 133.9 (2C), 132.2 (2C), 130.2 (4C), 129.5 (4C), 129.4 (2C), 129,2 (4C), 128.8 (2C), 128.2 (4C), 127.7 (2C), 127.5 (2C), 73.1 (2C), 63.1 (2C), 52.9 (2C), 51.5 (2C), 45.5 (2C), 40.2 (2C), 36.4 (2C); IR (cm^−1^) ν_max_: 3027, 2948, 1738, 1587, 1492, 1398, 1359, 1244, 1101, 1023, 704; ESIMS *m/z*: 1234 (21), 1233 (30), 1232 (47), 1231 (M^+^, 64), 1230 (100), 1228 (78), 1229 (49); anal. calcd for C_58_H_54_Cl_4_N_4_NiO_10_S_2_: C, 56.5; H, 4.4; N, 4.5; S, 5.2; found: C, 56.9; H, 4.2; N, 4.6; S, 5.1.

**{Dimethyl (2*RS*,4*SR*,5*RS*)-1-(benzoylcarbamothioyl)-2-benzyl-5-(2,4-dimethoxyphenyl)pyrrolidine-2,4-dicarboxylate}_2_Ni (L2-Ni):** Red solid, 109 mg, 90% yield; mp 305–307 °C (MeOH, decomp.); ^1^H NMR (400 MHz) δ 8.05 (d, *J* = 7.5 Hz, 4H), 7.67 (d, *J* = 8.6 Hz, 2H), 7.50–7.33 (m, 12H), 7.22 (d, *J* = 7.0 Hz, 4H), 6.40 (dd, *J* = 8.6 Hz, 2.3 Hz, 2H), 6.22 (d, *J* = 2.2 Hz, 2H), 5.48 (d, *J* = 9.6 Hz, 2H-5), 4.16 (d, *J* = 14.0 Hz, 2H-6), 3.78 (s, 6H), 3.73 (s, 6H), 3.55 (s, 6H), 3.39 (d, *J* = 14.1 Hz, 2H), 3.18 (s, 6H), 2.90–2.80 (m, 2H), 2.32–2.19 (m, 4H); ^13^C NMR (100 MHz) δ 173.6 (2CS), 173.4 (2CO), 172.9 (2CO), 170.3 (2CO), 160.2 (2C) , 158.0 (2C), 136.9 (2C), 136.3 (2C), 131.8 (2C), 130.3 (4C), 129.5 (4C), 129.0 (4C), 128.5 (2C), 128.0 (4C), 127.5 (2C), 118.5 (2C), 103.8 (2C), 98.5 (2C), 72.8 (2C), 60.6 (2C), 55.8 (2C), 55.1 (2C), 52.7 (2C), 51.5 (2C), 45.8 (2C), 40.4 (2C), 36.8 (2C); IR (cm^−1^) ν_max_: 3023, 2947, 1735, 1587, 1496, 1396, 1361, 1268, 1205, 1122, 1024, 703; ESIMS *m/z*: 1216 (5), 1215 (25), 1214 (M^+^, 38), 1213 (67), 1212 (100); anal. calcd for C_62_H_66_N_4_NiO_14_S_2_: C, 61.3; H, 5.4; N, 4.6; S, 5.3; found: C, 61.6; H, 5.7; N, 4.7; S, 5.0.

**{Dimethyl (2*RS*,4*SR*,5*RS*)-2-[(1*H*-indol-3-yl)methyl]-1-(benzoylcarbamothioyl)-5-(4-chlorophenyl)pyrrolidine-2,4-dicarboxylate}_2_Ni (L3-Ni):** Red solid, 100 mg, 81% yield; mp 221–223 °C (MeOH, decomp.); ^1^H NMR (400 MHz) δ 8.26 (s, 2H), 8.12–8.07 (m, 4H), 7.68–7.63 (m, 2H), 7.52–7.05 (m, 22H), 5.04 (d, *J* = 9.8 Hz, 2H-5), 4.29 (d, *J* = 15.1 Hz, 2H-6), 3.79 (s, 6H), 3.69 (d, *J* = 15.0 Hz, 2H), 3.06 (s, 6H), 2.92 (t, *J* = 12.8 Hz, 2H), 2.54–2.49 (m, 2H), 2.36 (dd, *J* = 12.9, 7.2 Hz, 2H); ^13^C NMR (100 MHz) δ 173.6 (2CS), 173.3 (2CO), 169.5 (2CO), 169.5 (2CO), 136.7 (2C), 135.9 (2C), 133.7 (2C), 133.3 (2C), 132.2 (2C), 129.8 (2C), 129.5 (4C), 128.2 (4C), 128.1 (2C), 128.0 (2C), 123.8 (2C), 122.5 (2C), 120.3 (2C), 118.6 (2C), 118.3 (2C), 111.5 (2C), 110.6 (2C), 110.3 (2C), 73.8 (2C), 67.4 (2C), 52.8 (2C), 51.5 (2C), 46.4 (2C), 36.9 (2C), 30.0 (2C); IR (cm^−1^) ν_max_: 3414, 3284, 3054, 2952, 1737, 1696, 1643, 1485, 1385, 1262, 1172, 1098, 740; ESIMS *m/z*: 1243 (17), 1242 (24), 1241 (42), 1240 (M^+^, 75%), 1239.2420 (67), 1238 (100); anal. calcd for C_62_H_58_Cl_2_N_6_NiO_10_S_2_: C, 60.0; H, 4.7; N, 6.7; S, 5.2; found: C, 60.5; H, 4.6; N, 6.5; S, 5.4.

**{Dimethyl (2*RS*,4*SR*,5*RS*)-1-(benzoylcarbamothioyl)-2-benzyl-5-(2,4-dichlorophenyl)pyrrolidine-2,4-dicarboxylate}_2_Pd (L1-Pd):** Brownish yellow solid, 99 mg, 78% yield; mp 187–189 °C (MeOH, decomp.); ^1^H NMR (400 MHz) δ 8.18–8.15 (m, 6H, minor and major), 8.01–7.94 (m, 4H, minor and major), 7.54–7.14 (m, 16H, minor and major), 5.56 (d, *J* = 9.9 Hz, 1H-5, major), 5.48 (d, *J* =10.1 Hz, 1H-5, minor), 4.28 (d, *J* = 14.0 Hz,1H-6, major), 4.23 (d, *J* = 14.1 Hz, 1H-6, minor), 3.80 (s, 3H, minor), 3.75 (s, 3H, major), 3.36–3.43 (m, 2H, major and minor), 3.22 (s, 3H, minor), 3.21 (s, 3H, major), 2.99–2.91 (m, 2H, major and minor), 2.35–2.28 (m, 2H, major and minor ), 2.26–2.16 (m, 2H, major and minor); ^13^C NMR (100 MHz) δ 173.1 (C=S minor), 173.0 (CS major), 172.2 (CO minor), 172.1 (CO major), 172.0 (CO minor), 172.05 (CO major), 169.7 (CO minor), 169.6 (CO major), 136.5 (minor), 136.4 (major), 136.3 (minor and major), 135.3 (major), 135.2 (minor), 134.1 (major), 134.0 (minor), 133.9 (minor and major), 132.2 (C major), 130.2 (3C minor), 130.1 (minor and major), 130.0 (4C minor), 129.6 (minor), 129.5 (major), 129.2 (3C major), 129.0 (minor), 128.8 (major), 128.2 (4C major), 127.9 (minor), 127.8 (major), 127.6 (2C minor), 73.5 (minor), 73.4 (major), 64.1 (minor), 63.9 (major), 53.1 (minor), 53.0 (major), 51.6 (major and minor), 45.9 (major), 45.5 (minor), 40.1 (minor), 40.0 (major), 36.8 (minor), 36.6 (major); IR (cm^−1^) ν_max_: 3027, 2948, 1737, 1497, 1396, 1361, 1246, 1101, 701; ESIMS *m/z*: 1283 (29), 1282 (46), 1281 (60), 1280 (96), 1279 (M^+^, 62%), 1278 (100), 1277 (81), 1276 (78), 1275 (66); anal. calcd for C_58_H_54_Cl_4_N_4_O_10_PdS_2_: C, 54.5; H, 4.3; N, 4.4; S, 5.0; found: C, 54.9; H, 4.0; N, 4.6; S, 4.7.

**{Dimethyl (2*RS*,4*SR*,5*RS*)-1-(benzoylcarbamothioyl)-2-benzyl-5-(2,4-dimethoxyphenyl)pyrrolidine-2,4-dicarboxylate}_2_Pd (L2-Pd):** Brownish yellow solid, 112 mg, 89% yield; mp 253–255 °C (MeOH, decomp.); ^1^H NMR (400 MHz) δ 8.18–8.15 (m, 4H, minor and major), 7.79 (d, *J* = 8.6 Hz, 1H, minor), 7.72 (d, *J* = 8.6 Hz, 1H, major), 7.51–7.29 (m, 12H, minor and major), 7.22–7.13 (m, 4H, minor and major), 6.47 (dd, *J* = 8.6 Hz, 2.4 Hz, 1H, minor), 6.40 (dd, *J* = 8.6 Hz, 2.4 Hz, 1H, major), 6.33 (d, *J* = 2.4 Hz, 1H, minor), 6.19 (d, *J* = 2.4 Hz, 1H, major), 5.55 (d, *J* = 9.1 Hz, 1H-5, major), 5.49 (d, 9.7 Hz, 1H-5, minor), 4.23 (d, *J* = 14.0 Hz, 1H-6, major), 4.17 (d, *J* = 14.1 Hz, 1H-6, minor), 3.81 (s, 3H, minor), 3.79 (s, 3H, major), 3.77 (s, 3H, major), 3.75 (s, 3H, minor), 3.74–3.64 (m, 2H, major and minor), 3.59 (s, 3H, minor), 3.58 (s, 3H, major), 3.42 (d, *J* = 14.0 Hz, 1H, major), 3.37 (d, *J* = 14.1 Hz, 1H, minor), 3.18 (s, 6H, minor and major), 2.31–2.24 (m, 4H, minor and major); ^13^C NMR (100 MHz) δ 173.4 (CS minor), 173.3 (CS major), 172.2 (CO major), 172.1 (CO minor), 171.6 (CO major), 171.5 (CO minor), 170.3 (CO minor), 170.2 (CO major), 160.3 (major), 160.2 (minor), 158.0 (major), 157.9 (minor), 136.8 (minor), 136.7 (major), 136.71 (minor), 136.6 (major), 131.9 (major), 131.8 (minor), 130.4 (minor), 130.3 (major), 130.2 (major), 130.1 (minor), 130.0 (2C major and minor), 129.0 (2C major and minor), 128.6 (major), 128.6 (minor), 128.1 (2C major and minor), 127.5 (major), 127.4 (minor), 118.9 (major), 118.5 (minor), 103.9 (minor), 103.8 (major), 98.7 (major), 98.5 (minor), 73.2 (major), 73.1 (minor), 61.6 (major), 61.5 (minor), 55.9 (minor), 55.8 (major), 55.2 (major), 55.1 (minor), 52.8 (major), 52.8 (minor), 51.5 (major), 51.4 (minor), 45.8 (minor), 45.7 (major), 40.4 (minor), 40.4 (major), 37.1 (minor), 37.0 (major); IR (cm^–1^) ν_max_: 3055, 2949, 1735, 1698, 1587, 1504, 1394, 1357, 1258, 1206, 1099, 1030, 702; ESIMS *m/z*: 1266 (4), 1265 (28), 1264 (49), 1263 (69), 1262 (96), 1261 (M^+^, 68), 1260 (100), 1259 (87%), 1258 (48%); anal. calcd for C_62_H_66_N_4_O_14_PdS_2_: C, 59.0; H, 5.3; N, 4.4; S, 5.1; found: C, 59.5; H, 5.3; N, 4.1; S, 4.8.

**{Dimethyl (2*RS*,4*SR*,5*RS*)-2-[(1*H*-indol-3-yl)methyl]-1-(benzoylcarbamothioyl)-5-(4-chlorophenyl)pyrrolidine-2,4-dicarboxylate}_2_Pd (L3-Pd):** Brownish yellow solid, 100 mg, 78% yield; mp 217–219 °C (MeOH, decomp.). ^1^H NMR (400 MHz) δ 8.26 (d, *J* = 1.9 Hz, 2H), 8.21–8.15 (m, 4H,), 7.63 (d, *J* = 7.9 Hz, 2H), 7.52–7.39 (m, 6H), 7.29–7.25 (m, 6H), 7.23–7.18 (m, 4H), 7.15–7.11 (m, 4H), 7.02 (d, *J* = 2.2 Hz, 2H), 5.14 (d, *J* = 9.8 Hz, 2H-5), 4.28 (d, *J* = 15.0 Hz, 2H-6), 3.87 (s, 6H), 3.67 (d, *J* = 14.7 Hz, 2H), 3.11 (s, 6H), 2.94 (t, *J* = 12.8 Hz, 2H), 2.52 (ddd, *J* = 12.8, 9.8, 7.3 Hz, 2H), 2.40 (dd, *J* = 12.9, 7.2 Hz, 2H); ^13^C NMR (100 MHz) δ 173.3 (CS major), 173.2 (CS minor) 172.3 (CO major and minor), 172.1 (CO major), 172.0 (CO minor), 169.6 (CO major), 169.4 (CO minor), 136.7 (minor and major), 136.5 (minor), 136.4 (major), 135.9 (minor), 135.8 (major), 133.8 (minor), 133.4 (major), 132.2 (minor and major), 130.0 (4 minor and major), 129.8 (3× minor and major), 128.2 (minor and major), 128.1 (minor), 128.0 (major), 123.9 (minor), 123.8 (major), 122.5 (minor), 122.4 (major), 120.4 (minor), 120.3 (major), 118.6 (minor), 118.3 (major), 111.5 (major), 111.4 (minor), 110.6 (minor), 110.2 (major), 74.2 (major), 73,8 (minor), 68.3 (major), 68.2 (minor), 52.9 (major), 52.9 (minor), 51.5 (major), 51.4 (minor), 46.5 (minor), 46.4 (major), 37.2 (major), 36.9 (minor), 30.1 (major), 30.0 (minor); IR (cm^−1^) ν_max_: 3417, 3284, 3053, 2954, 1735, 1698, 1643, 1485, 1433, 1384, 1263, 1172, 1098, 741; ESIMS *m/z*: 1293.2088 (18.4%), 1292.2109 (13.7%), 1291.2076 (41.5%), 1290.2042 (61.9%), 1289.2105 (64.9%), 1288.2072 (M^+^, 96.8%), 1287.2101 (67.1%), 1286.2068 (100.0%), 1285.2084 (81.7%), 1284.2073 (40.8%); anal. calcd for C_62_H_58_Cl_2_N_6_O_10_PdS_2_: C, 57.9; H, 4.5; N, 6.5; S, 5.0%; found: C, 58.4; H, 4.3; N, 6.6; S, 5.1.

### Biological tests

The antibacterial activity of the complexes against standard bacterial strains (*Staphylococcus aureus* [ATCC 25925], *Bacillus subtilis* [ATCC 6633], *Aeromonas hydrophila* [ATCC 95080], *Escherichia coli* [ATCC 25923], and *Acinetobacter baumannii* [ATCC 02026]) was determined with a resazurin microtitre assay (REMA). These standard strains were obtained from Refik Saydam Hıfzıssıhha Institute (Ankara, Turkey). To obtain an initial concentration of 1000 μg/mL, the novel synthesized compounds were dissolved in DMSO to prepare stock solutions and then, 0.22 μm membrane filters were used to sterilization of the compounds. Serial two-fold dilutions of the compounds and ampicillin (standard reference drug) were prepared and the concentrations of the substances to be tested were adjusted to 500–0.24 μg/mL. All antibacterial activity determinations were repeated twice [28].

Moreover, in order to determine the minimum inhibitory concentration (MIC) values of synthesized novel compounds against *M. tuberculosis* H37Rv standard strain, REMA method was used [29–30]. H37Rv standard strain was provided from Refik Saydam National Public Health Agency, the National Tuberculosis Reference Laboratory (Ankara). Rifampicin (RIF) (Sigma R3501) and isoniazid (INH) (Sigma I3377) were used as reference drugs. To obtain an initial concentration of 1000 μg/mL, stock solutions of the compounds were dissolved in DMSO. In a 96-well microtiter plate, a two-fold dilution series of the compounds and reference drugs were prepared in 100 μL of 7H9-S medium. A 100 μL of H37Rv standard strain working solution was added in microtiter plate wells and then, 250–0.12 μg/mL final concentration ranges of the compounds were obtained. In each anti-TB activity determination, a sterility control (without H37Rv working solution) and a growth control (containing no antibiotics) were included in each plate. Effects of DMSO were controlled by inoculated broth supplement at the same solutions.

Microdilution broth method [31–32] was used to determine the MIC values of the target compounds against to *Candida albicans* (ATCC 14053), *C. tropicalis* (ATCC 1369) and *C. glabrata* (ATCC 15126) standard strains (which were provided from Refik Saydam Hıfzıssıhha Institute in Ankara, Turkey) with respect to the standard document (M27-A2) of NCCLS [33]. As a reference antifungal agent fluconazole (Sigma, F8929) was used (Table 2). As mentioned in the M27-A2 document, experiments were performed in RPMI 1640 medium adjusted to pH 7.0 with 0.165 M 3-(*N*-morpholino)-propane sulfonic acid (MOPS, Sigma, M1254). With dissolving the tested compounds in DMSO, an initial concentration of 1000 μg/mL was prepared. After this, serial twofold dilutions of the compounds and fluconazole in 100 μL of RPMI 1640 medium were prepared in the plates. Working suspensions of the standard *Candida* strains were prepared according to the M27-A2 document. After adding 100 μL of working suspension in microtiter plate wells, 250–0.12 μg/mL final concentration ranges were obtained. Inoculated plates were incubated for 48 h in ambient air at 35 °C. The lowest concentration of a compound that inhibits growth of the standard *Candida* strain was determined as the MIC value, which detected visually. Fluconazole (Sigma, F8929) showed activity with a range of 3.90–31.25 μg/mL when tested against the indicated yeast.

## Supporting Information

File 1 General procedure for the synthesis of ligands (**L1–L3**) and complexes, NMR spectra and computational data.
