# Supplementary material for: Biological properties and conformational studies of amphiphilic Pd(II) and Ni(II) complexes bearing functionalized aroylaminocarbo-N-thioylpyrrolinate units
Source: Beilstein J Org Chem. 2021 Dec 2;17:2812–21. doi: 10.3762/bjoc.17.192 (PMC8649204; doi:10.3762/bjoc.17.192)
Supplement: File 1 — General procedure for the synthesis of ligands (L1–L3) and complexes, NMR spectra and computational data. [file Beilstein_J_Org_Chem-17-2812-s001.pdf]

BEILSTEIN JOURNAL OF ORGANIC CHEMISTRY

Supporting Information

for

Biological properties and conformational studies of amphiphilic Pd(II) and Ni(II) complexes bearing functionalized aroylaminocarbo-*N*-thioylpyrrolinate units

Samet Poyraz, Samet Belveren, Sabriye Aydınoğlu, Mahmut Ulger, Abel de Cózar, Maria de Gracia Retamosa, Jose M. Sansano and H. Ali Döndaş

Beilstein J. Org. Chem. **2021,** *17,* 2812–2821. doi:10.3762/bjoc.17.192

General procedure for the synthesis of ligands (L1–L3) and complexes, NMR spectra and computational data

License and Terms: This is a supporting information file under the terms of the Creative Commons Attribution License (https://creativecommons.org/licenses/by/4.0). Please note that the reuse, redistribution and reproduction in particular requires that the author(s) and source are credited and that individual graphics may be subject to special legal provisions.
The license is subject to the *Beilstein Journal of Organic Chemistry* terms and conditions: (https://www.beilstein-journals.org/bjoc/terms)

Table of contents:

1. **General information S2**
2. **General procedure for the synthesis of ligands (L1–L3) and complexes S2**
3. **NMR spectra S3**
4. **Computational data S10**
5. **Reference S29**

1. General information

All commercially available reagents and solvents were used without further purification. Flash column chromatography was performed using silica gel 60 (230–400 mesh). Kieselgel columns were packed with silica gel GF254 (Merck 7730). Flash chromatography was carried out on handpacked columns of Merck silica gel 60 (0.040–0.063 mm). Melting points were determined on a Stuart SMP3 hot stage apparatus. The structurally most important peaks of the IR spectra (recorded using a Nicolet 510 P-FT) are listed and wave numbers are given in cm^{-1}. Nuclear magnetic resonance spectra and decoupling experiments were determined at 250 MHz on a Q.E 300 instrument, at 300 MHz on a Bruker Avance AC-300 and at 500 MHz on a Bruker AM500 spectrometer as specified. Chemical shifts are given in parts per million (δ) downfield from tetramethylsilane as internal standard. Spectra were determined in $CDCl_3$.The following abbreviations are used to describe peak patterns where appropriate: s = singlet, d = doublet, t = triplet, q = quartet, m = multiplet. All coupling constants (J) are given in Hz and chemical shifts in ppm. Low-resolution electron impact (EI) mass spectra were obtained at 70 eV using a Shimadzu QP-5000 by injection or DIP; fragment ions in m/z are given with relative intensities (%) in parentheses. High-resolution mass spectra (HRMS) were measured on an instrument using a quadrupole time-of-flight mass spectrometer (QTOF) and also through the electron impact mode (EI) at 70 eV using a Finnigan VG Platform or a Finnigan MAT 95S. VCD analysis was recorded with a Jasco FVS-6000. Microanalyses were measured in a CHNS apparatus with a Micro TruSpec from LECO detection system.

2. General procedure for the novel compounds

Synthesis of selected ligands (L1-L3)

The known pyrrolidines and aroylaminocarbo-N-thioylpyrrolidine compounds (**L1–L3**) were synthesized according to published procedures [1] **.**

Preparation of Ni(II), and Pd(II) complexes

A solution of metal acetate (0.1 mmol) in methanol (10 mL) was added dropwise to the solution of aroylaminocarbo-*N*-thioylpyrrolidine ligands (0.2 mmol) in methanol (25 mL) at room temperature. The resulting mixture was stirred for 48 h and the precipitated complexes were filtered and washed with methanol.

3. NMR spectra

^{1}H NMR spectrum of L1-Ni ($CDCl_3$, 400 MHz)

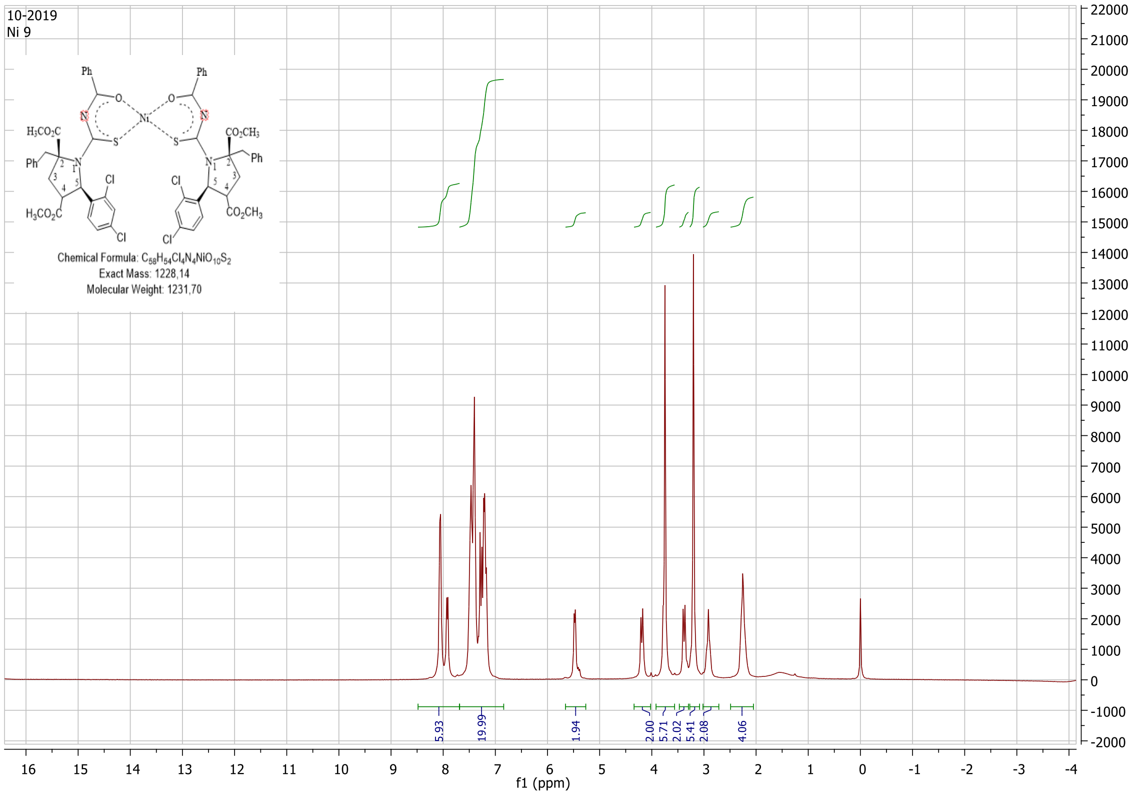

^{13}C NMR spectrum of L1-Ni ($CDCl_3$, 100 MHz)

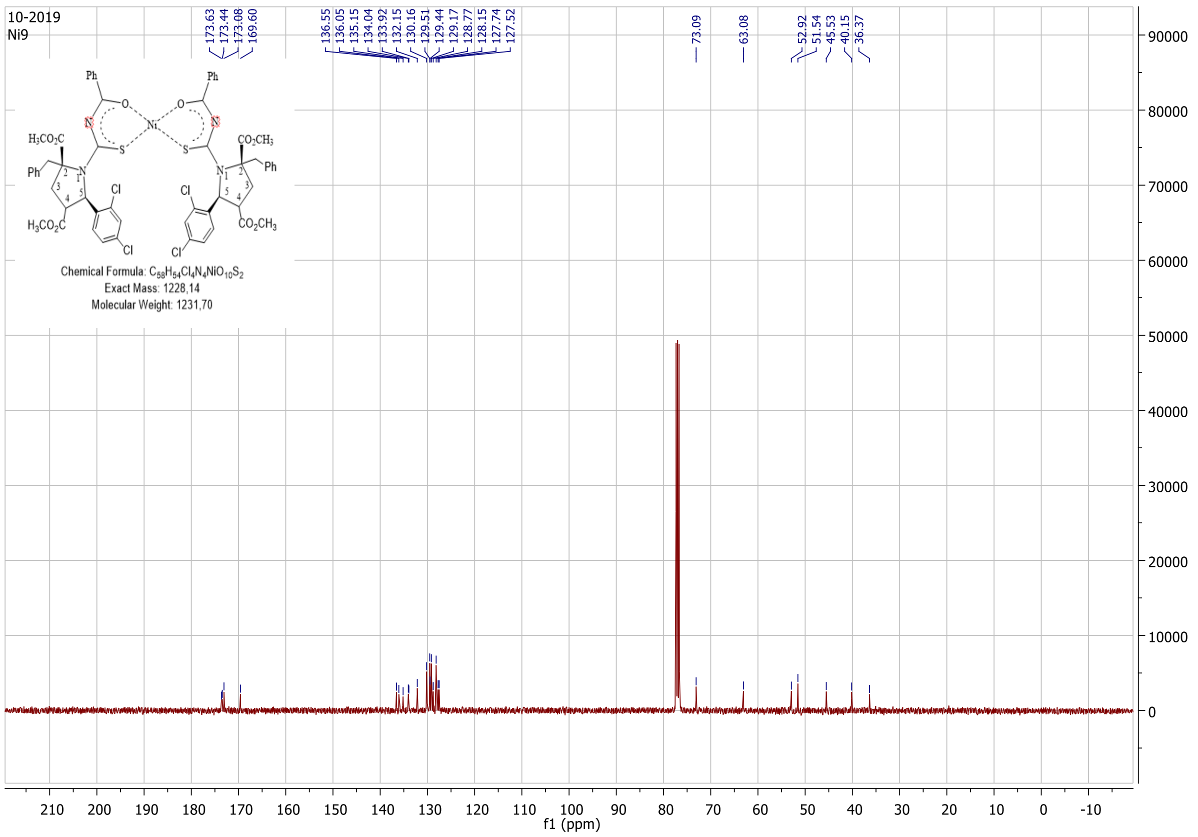

^{1}H NMR spectrum of L2-Ni ($CDCl_3$, 400 MHz)

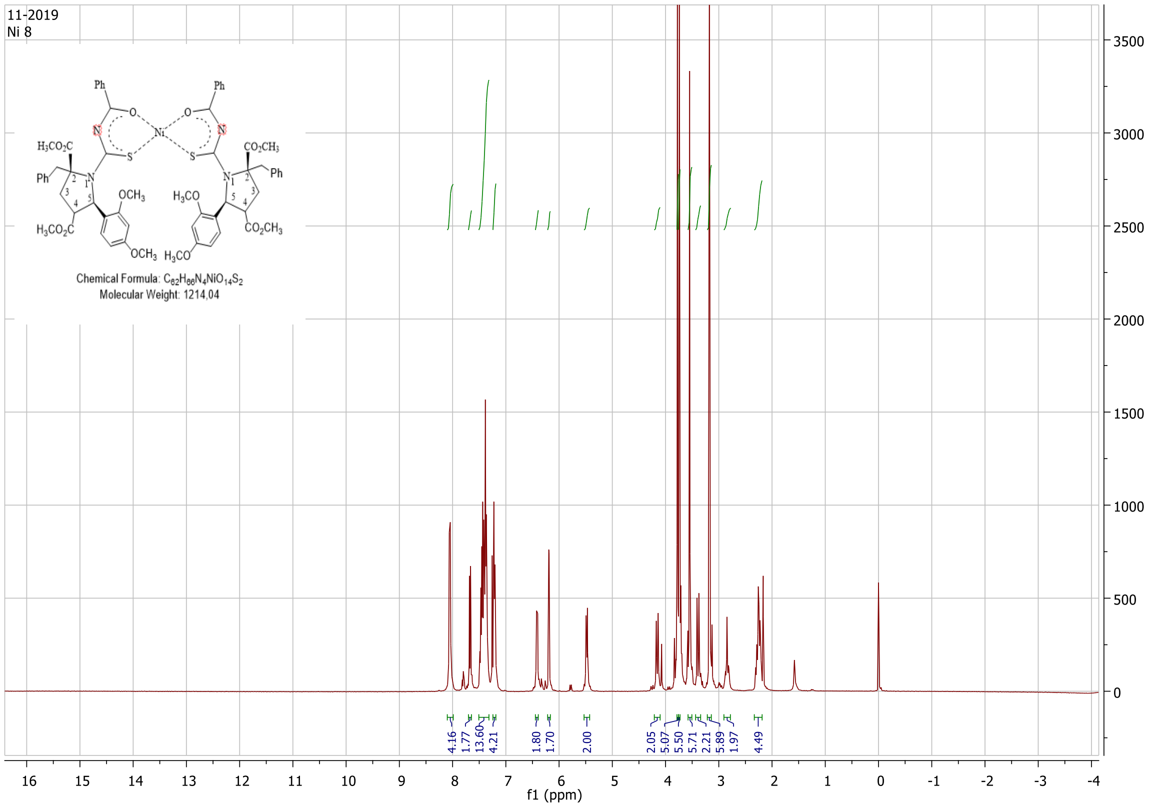

^{13}C NMR spectrum of L2-Ni ($CDCl_3$, 100 MHz)

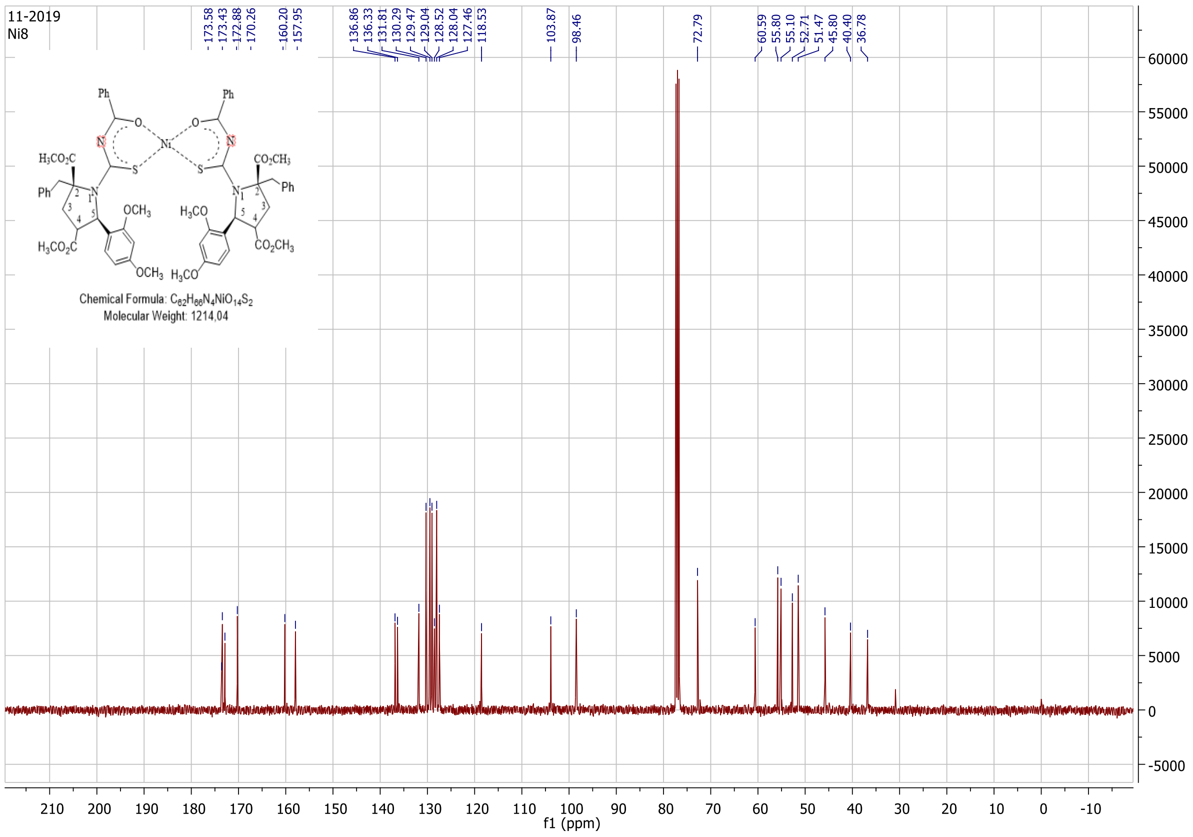

^{1}H NMR spectrum of L3-Ni ($CDCl_3$, 400 MHz)

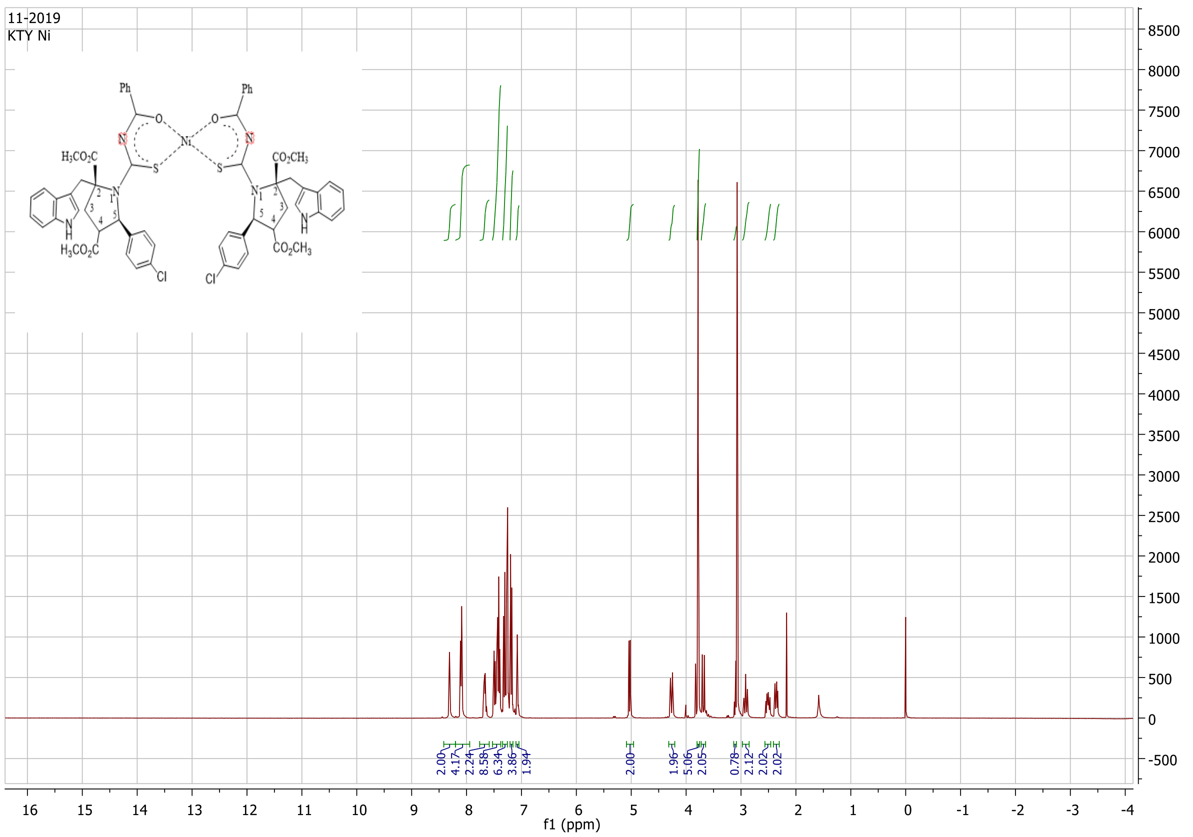

^{13}C NMR spectrum of L3-Ni ($CDCl_3$, 100 MHz)

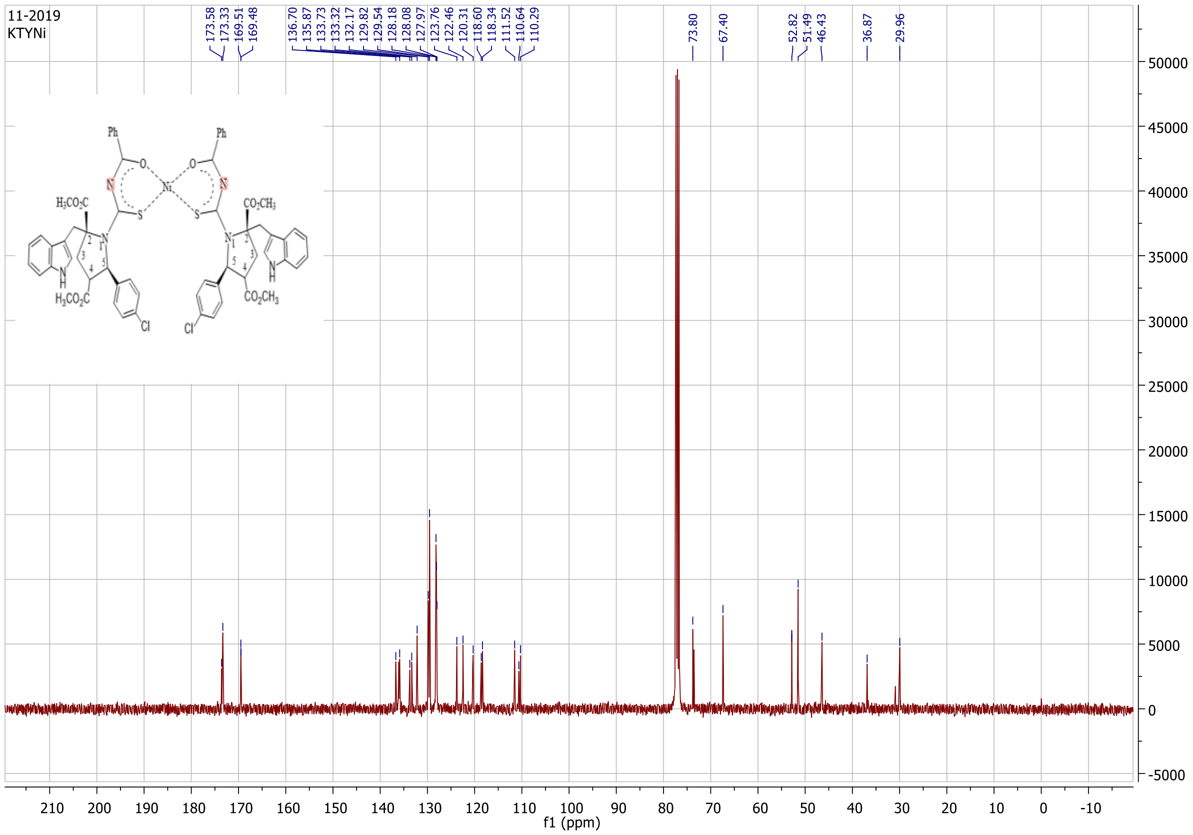

^{1}H NMR spectrum of L1-Pd ($CDCl_3$, 400 MHz)

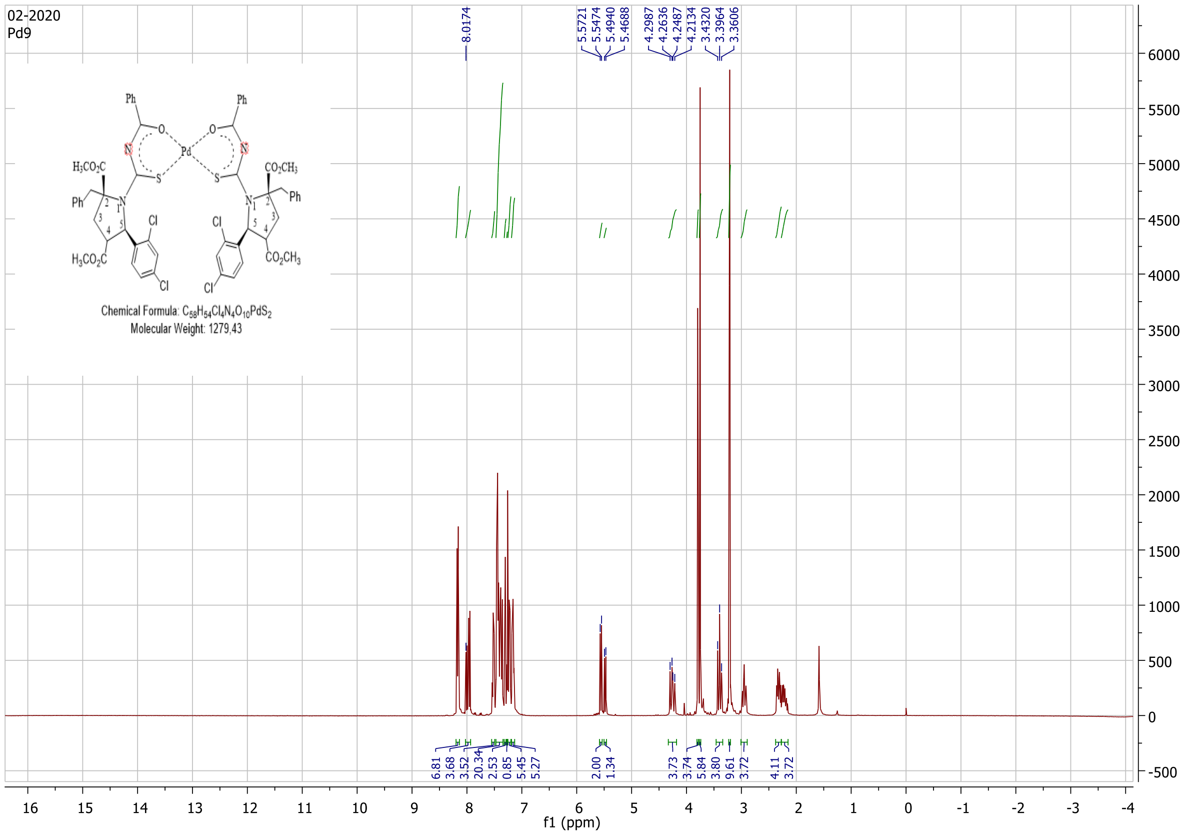

^{13}C NMR spectrum of L1-Pd ($CDCl_3$, 100 MHz)

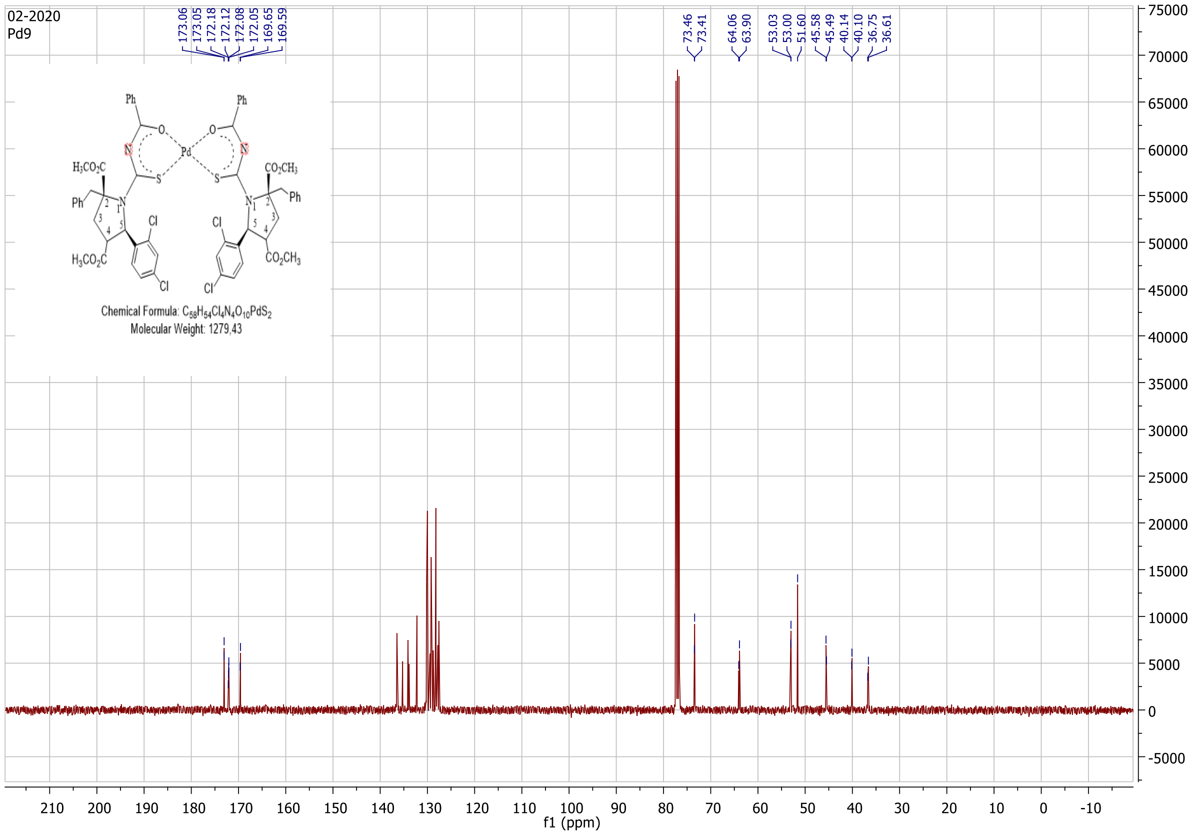

DEPT-135 NMR spectrum of L1-Pd (CDCl3, 100 MHz)

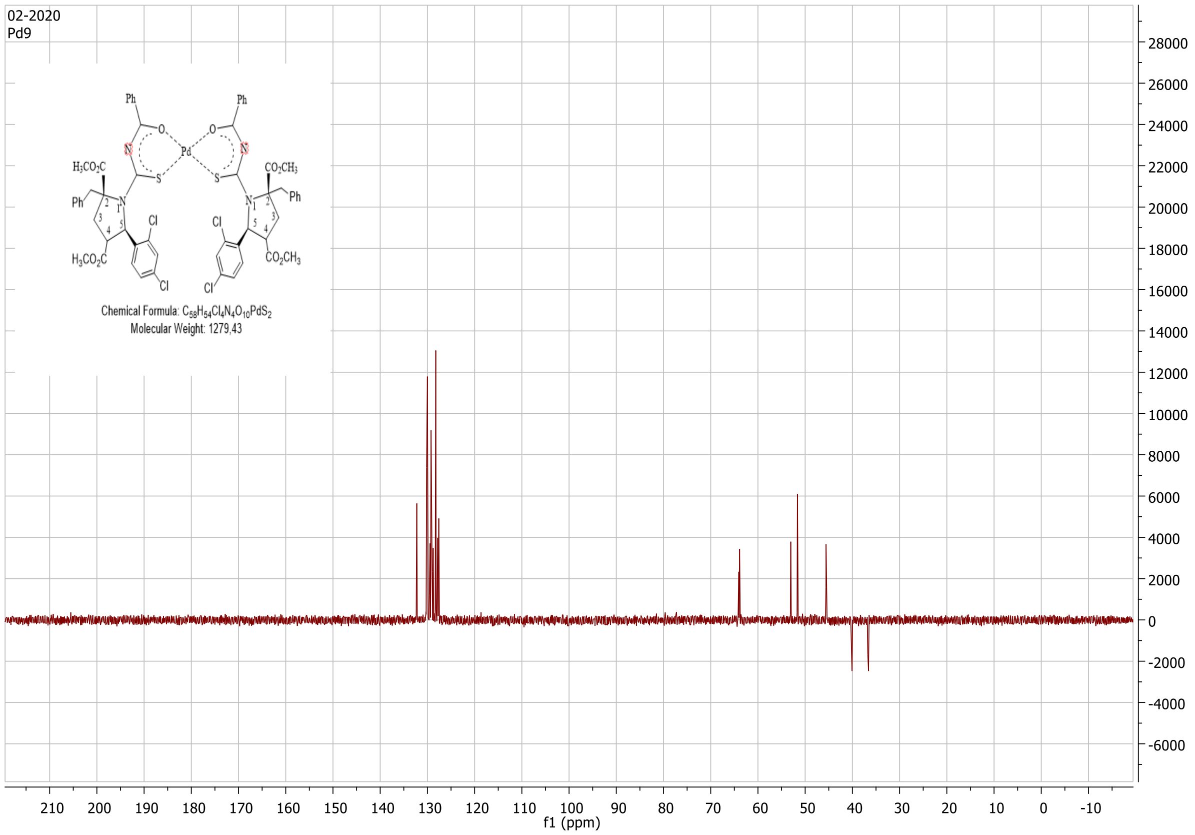

^{1}H NMR spectrum of L2-Pd (CDCl3, 400 MHz)

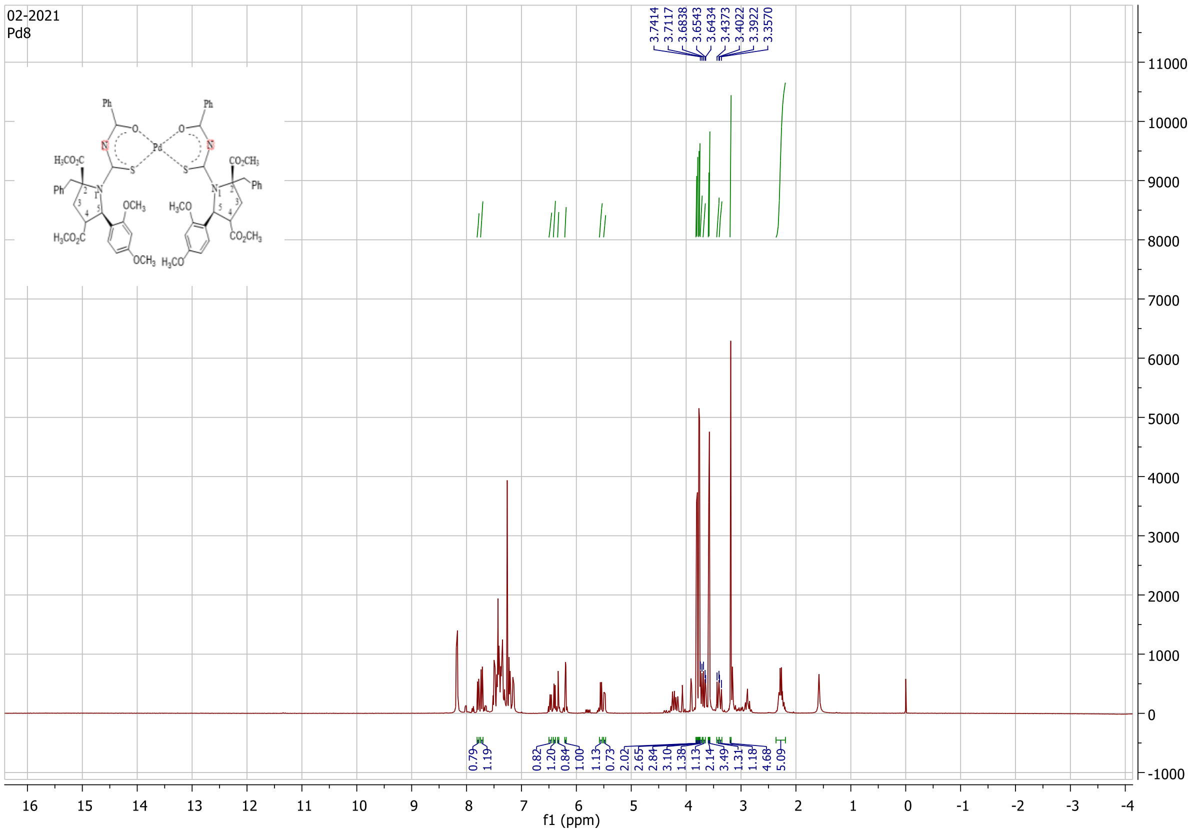

^{13}C NMR spectrum of L2-Pd ($CDCl_3$, 100 MHz)

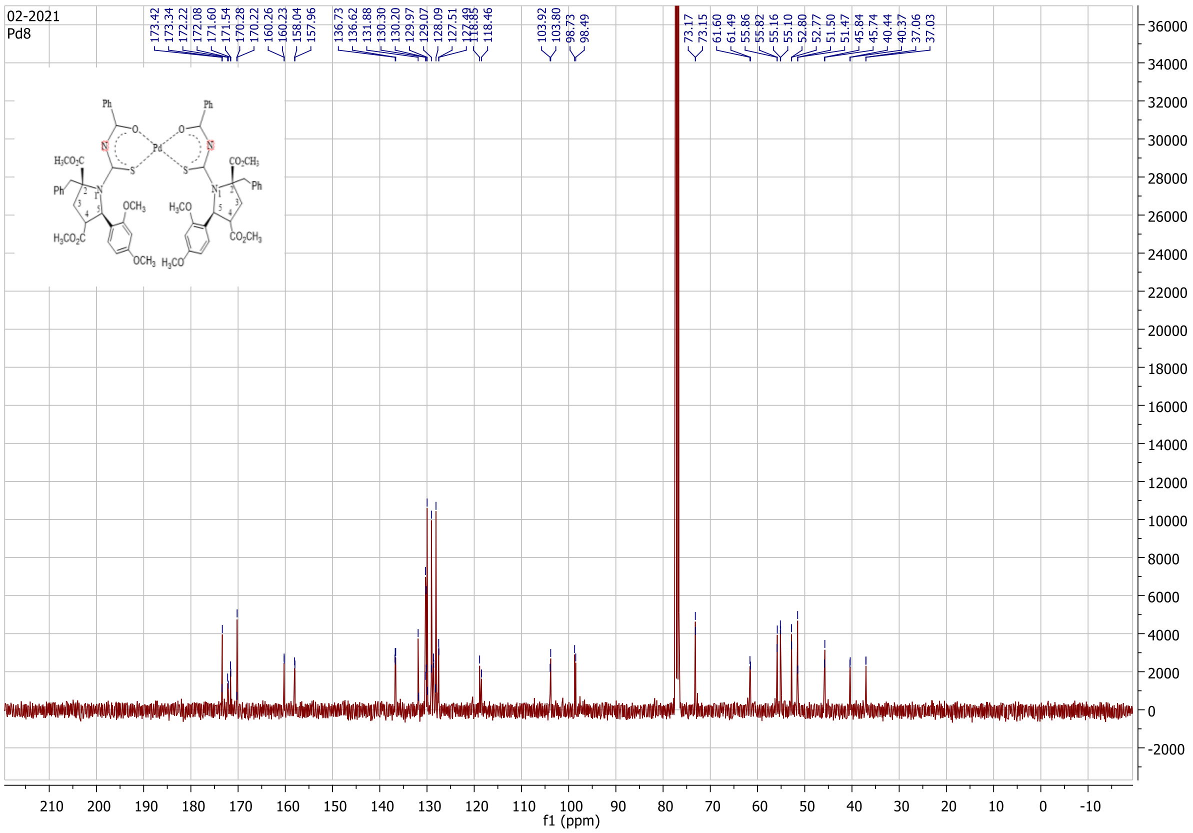

DEPT-135 NMR spectrum of L2-Pd ($CDCl_3$, 100 MHz)

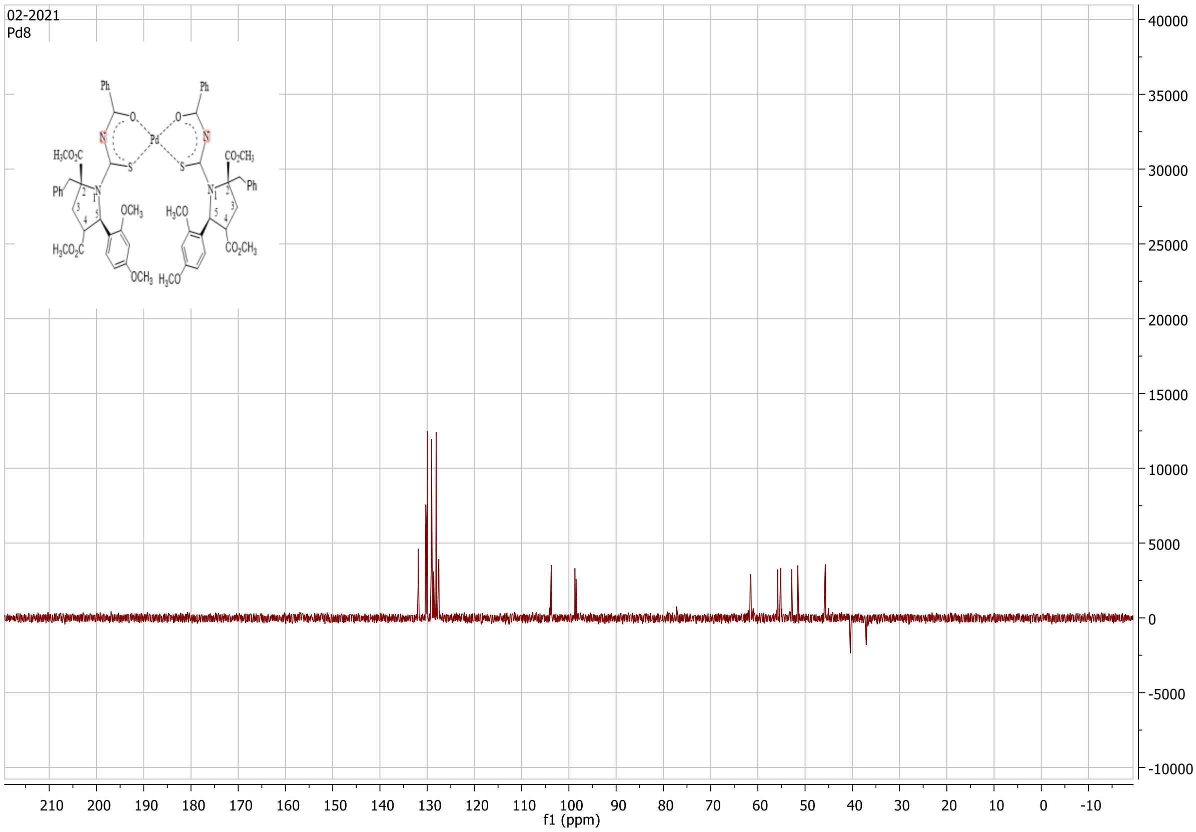

^{1}H NMR spectrum of L3-Pd ($CDCl_3$, 400 MHz)

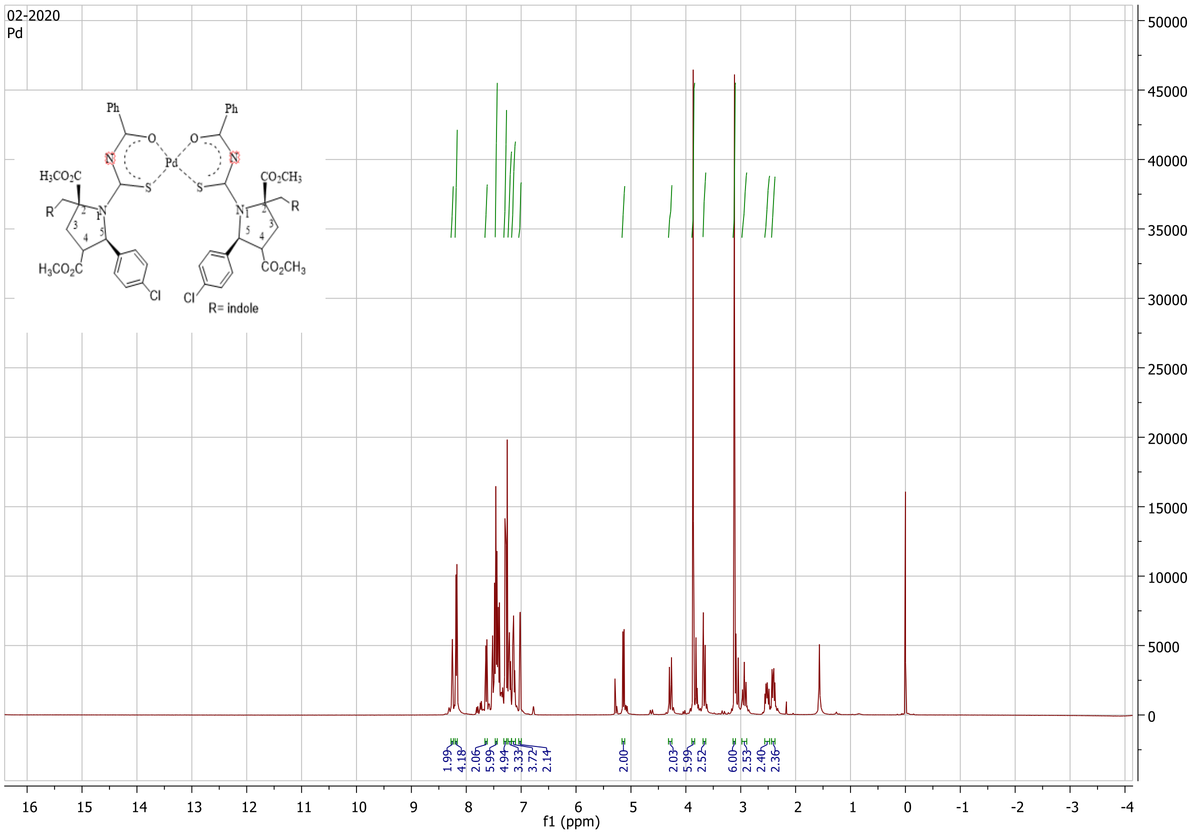

^{13}C NMR spectrum of L3-Pd ($CDCl_3$, 100 MHz)

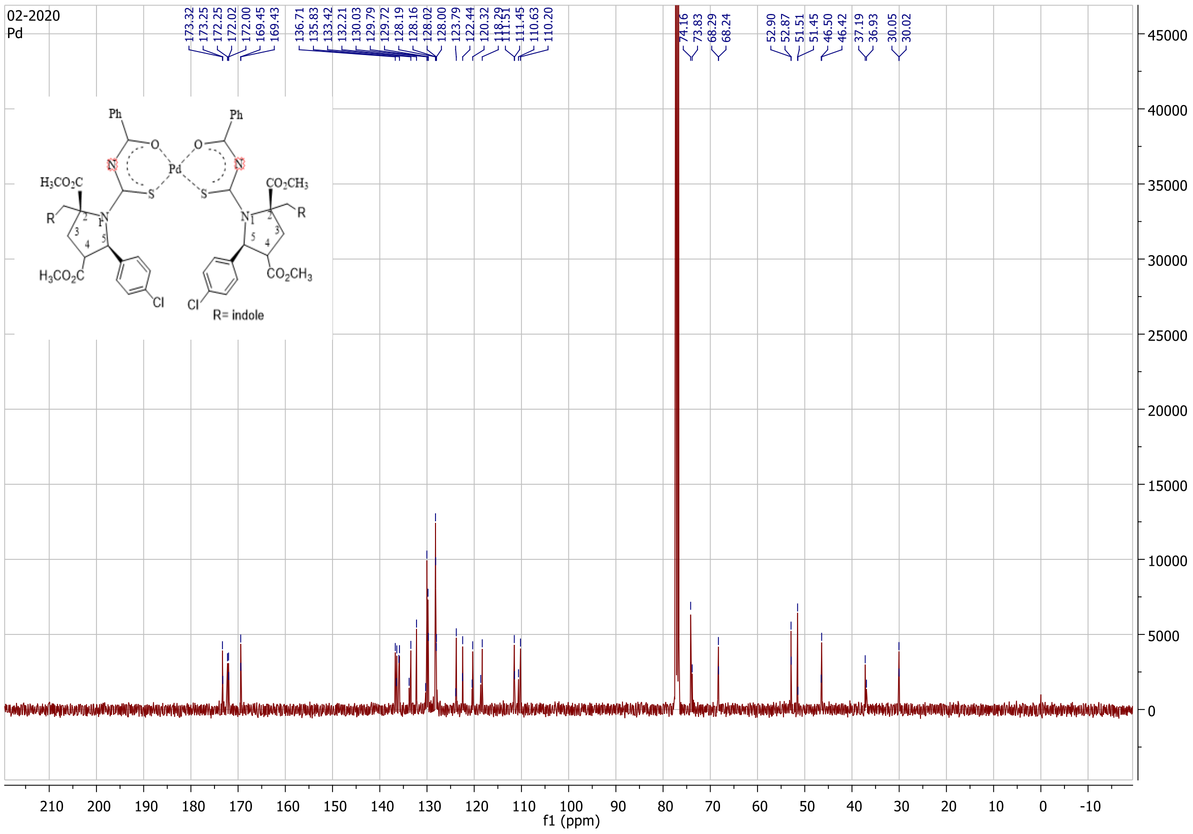

4. Computational data

Table S1. Total electronic energies[a] (E, in a.u.), zero point correction of the energy[a] (ZPCE), thermal corrections to Gibbs free energies[a] (TCGFE, in a.u.), and number of imaginary frequencies (NIMAG) of all stationary points discussed in the main text computed at B3LYP-GD3BJ/TZVP and B3LYP-GD3BJ/TZVP&SDD for ligands and metallic complexes, respectively.

Structure	E	ZPCE	TCGFE	NIMAG(υ)
**L1-A**	-2925.789788	0.501391	0.428269	0
**L1-B**	-2925.792137	0.501391	0.429011	0
**NiL1-A**	-6021.426290	0.983766	0.865176	0
**NiL1-B**	-6021.411591	0.982983	0.863167	0
**PdL1-A**	-5978. 383999	0.982877	0.863245	0
**PdL1-B**	-5978.371128	0.982326	0.858637	0
**L3-A**	-2597.793045	0.540144	0.466631	0
**L3-B**	-2597.796054	0.539892	0.467794	0
**NiL3-A**	-5365.427144	1.060778	0.936607	0
**NiL3-B**	-5365.402703	1.059792	0.935522	0
**PdL3-A**	-5322.387996	1.060188	0.938625	0
**PdL3-B**	-5322.373023	1.059147	0.932786	0

[a]Computed at 298.15 K.

Cartesian coordinates (optimized at B3LYP-GD3BJ/TZVP or B3LYP-GD3BJ/TZVP&SDD level) of all the stationary points collected in the main text

L1-A

```
 ---------------------------------------------------------------------
 Center     Atomic      Atomic             Coordinates (Angstroms)
 Number     Number       Type             X           Y           Z
 ---------------------------------------------------------------------
      1          6           0        0.842667    3.061126   -1.214102
      2          6           0        0.944284    3.125730    0.177397
      3          6           0        0.378426    4.216234    0.841643
      4          6           0       -0.307525    5.200180    0.137601
      5          6           0       -0.430809    5.109425   -1.245691
      6          6           0        0.154265    4.041476   -1.919941
      7          6           0        1.608352    2.021282    0.954667
      8          6           0        0.673066    0.807963    1.235127
      9          7           0        0.169719    0.193604   -0.022403
     10          6           0       -1.267963    0.430541   -0.255517
     11          6           0       -1.665630    1.422640    0.874258
     12          6           0       -0.630313    1.205011    1.965635
     13          6           0        0.921560   -0.308630   -1.028272
     14         16           0        0.326139   -0.617947   -2.561823
     15          6           0       -3.082932    1.213926    1.366269
     16          8           0       -3.393027    0.606701    2.365272
     17          6           0        1.376612   -0.205961    2.144679
     18          8           0        1.501000   -1.390980    1.909282
     19          7           0        2.233468   -0.622172   -0.667784
     20          6           0        3.376488   -0.360512   -1.398137
     21          6           0        4.597800   -1.054589   -0.893808
     22          6           0        4.539699   -2.262053   -0.192045
     23          6           0        5.709859   -2.872660    0.242562
     24          6           0        6.942514   -2.279435   -0.012472
     25          6           0        7.005434   -1.077633   -0.714213
     26          6           0        5.839241   -0.471003   -1.158534
```

27	8	0	3.401669	0.415484	-2.338270
28	8	0	-3.959609	1.778811	0.532126
29	6	0	-5.358719	1.554554	0.811297
30	8	0	1.794953	0.375232	3.258226
31	6	0	2.434696	-0.476824	4.242330
32	1	0	-0.478517	2.084628	2.584664
33	1	0	-0.962354	0.396927	2.616854
34	1	0	-1.596449	2.428191	0.467984
35	1	0	-1.376824	0.913675	-1.220576
36	1	0	3.314243	-0.946524	3.807434
37	1	0	2.711224	0.187027	5.054053
38	1	0	1.734175	-1.236058	4.583518
39	1	0	-5.895719	2.067469	0.020254
40	1	0	-5.574881	0.487465	0.790970
41	1	0	-5.619809	1.966496	1.784312
42	1	0	1.942392	2.391943	1.921587
43	1	0	2.489222	1.664067	0.424147
44	1	0	0.077676	3.971831	-2.997722
45	1	0	0.469560	4.292603	1.918410
46	1	0	3.591094	-2.748895	-0.006238
47	1	0	5.874929	0.460032	-1.707649
48	1	0	5.658660	-3.813123	0.775464
49	1	0	7.963494	-0.615407	-0.913853
50	1	0	7.852589	-2.754489	0.330899
51	1	0	1.302496	2.242167	-1.751692
52	1	0	-0.969273	5.871093	-1.795155
53	1	0	-0.745347	6.035856	0.668811
54	1	0	2.323208	-1.119053	0.211034
55	6	0	-2.059555	-0.859135	-0.262628
56	6	0	-3.158837	-1.046923	-1.100819
57	6	0	-1.740476	-1.894319	0.616298
58	6	0	-3.930508	-2.201201	-1.058930
59	6	0	-2.487596	-3.060315	0.680540
60	1	0	-0.871692	-1.797553	1.251431
61	6	0	-3.584211	-3.194675	-0.157765
62	1	0	-4.774240	-2.320462	-1.721917
63	1	0	-2.220955	-3.848741	1.369497
64	17	0	-4.556042	-4.663898	-0.091191
65	17	0	-3.612115	0.172054	-2.292558

L1-B

Center Number	Atomic Number	Atomic Type	Coordinates (Angstroms) X	Y	Z
1	6	0	-4.081543	1.327757	1.303079
2	6	0	-2.844052	1.237261	1.943236
3	6	0	-2.728912	0.514453	3.132360
4	6	0	-3.844033	-0.113175	3.673970
5	6	0	-5.071090	-0.037551	3.022426
6	6	0	-5.188343	0.683287	1.835983
7	6	0	-1.679607	1.913360	1.302884
8	8	0	-1.784208	2.930070	0.644067
9	7	0	-0.480229	1.233590	1.469790
10	6	0	0.745380	1.606490	0.897085
11	16	0	1.436377	3.095807	1.210313
12	7	0	1.294847	0.652149	0.123845
13	6	0	2.651419	0.747210	-0.458175
14	6	0	2.657188	-0.401607	-1.490334
15	6	0	1.666219	-1.434516	-0.970634
16	6	0	0.601777	-0.622434	-0.179285
17	6	0	3.747420	0.596214	0.626305
18	6	0	3.625948	-0.669877	1.430650
19	6	0	2.735144	-0.746881	2.504666
20	6	0	2.557194	-1.938010	3.199690
21	6	0	3.282158	-3.070738	2.840588
22	6	0	4.197450	-2.997637	1.794772
23	6	0	4.368382	-1.805263	1.098916
24	6	0	2.775421	2.062817	-1.253777
25	8	0	3.963079	2.640914	-1.101534
26	6	0	4.177575	3.863162	-1.840318
27	6	0	1.041280	-2.274003	-2.065357
28	8	0	0.352701	-3.289369	-1.534554
29	6	0	-0.414532	-4.103801	-2.447725

30	8	0	1.125777	-2.057101	-3.251204
31	8	0	1.926569	2.442916	-2.028016
32	1	0	3.655138	-0.814072	-1.614240
33	1	0	2.319418	-0.035300	-2.457111
34	1	0	2.146964	-2.113424	-0.270778
35	1	0	0.411191	-1.154182	0.748467
36	1	0	3.460027	4.616406	-1.518638
37	1	0	5.190215	4.170680	-1.600627
38	1	0	4.071214	3.682633	-2.908366
39	1	0	-0.906051	-4.845961	-1.827319
40	1	0	-1.150123	-3.488320	-2.963378
41	1	0	0.242747	-4.581162	-3.171934
42	1	0	4.709190	0.628871	0.118222
43	1	0	3.694895	1.464798	1.279358
44	1	0	1.857027	-1.979839	4.024559
45	1	0	5.080127	-1.757757	0.283464
46	1	0	-1.780614	0.459459	3.651850
47	1	0	-4.158126	1.886452	0.380410
48	1	0	-3.754098	-0.662032	4.602239
49	1	0	-6.141510	0.739498	1.326490
50	1	0	-5.936085	-0.537156	3.439539
51	1	0	2.183585	0.135352	2.804904
52	1	0	3.143594	-3.999461	3.379245
53	1	0	4.777695	-3.869222	1.519137
54	1	0	-0.560754	0.283951	1.803659
55	6	0	-0.708202	-0.401648	-0.905083
56	6	0	-1.866253	-1.101749	-0.566282
57	6	0	-0.795857	0.527065	-1.943224
58	6	0	-3.070046	-0.902859	-1.227178
59	6	0	-1.984148	0.748371	-2.621976
60	1	0	0.076829	1.112473	-2.200621
61	6	0	-3.110220	0.027251	-2.252423
62	1	0	-3.955105	-1.444173	-0.929594
63	1	0	-2.035383	1.478228	-3.416939
64	17	0	-4.638242	0.313791	-3.080030
65	17	0	-1.870957	-2.245964	0.777584

NiL1-A

Center Number	Atomic Number	Atomic Type	Coordinates (Angstroms) X	Y	Z
1	6	0	-4.027960	6.559911	-2.342457
2	6	0	-4.872892	5.496774	-2.036858
3	6	0	-4.351888	4.319700	-1.517369
4	6	0	-2.977372	4.196392	-1.299768
5	6	0	-2.132830	5.266585	-1.608136
6	6	0	-2.657411	6.441696	-2.127126
7	6	0	-2.393908	2.943589	-0.751449
8	8	0	-1.144559	2.933491	-0.576285
9	28	0	-0.000099	1.545237	-0.000345
10	16	0	-1.373871	-0.028626	-0.686800
11	6	0	-2.886792	0.715608	-0.216658
12	7	0	-3.249988	1.970283	-0.446254
13	7	0	-3.816879	-0.067188	0.360668
14	6	0	-3.588006	-1.473913	0.732281
15	6	0	-4.982722	-1.938474	1.255546
16	6	0	-5.747185	-0.655035	1.567466
17	6	0	-5.187599	0.415897	0.612880
18	6	0	-4.870985	-2.846300	2.463455
19	8	0	-4.503674	-4.081452	2.088838
20	6	0	-4.197210	-5.001042	3.151525
21	6	0	-5.106281	1.742592	1.388099
22	8	0	-5.989726	2.651711	0.955842
23	6	0	-5.957839	3.919463	1.639899
24	6	0	-5.983106	0.539582	-0.711939
25	6	0	-6.022136	-0.737697	-1.507445
26	6	0	-7.121490	-1.594637	-1.434281
27	6	0	-7.122531	-2.814477	-2.102073
28	6	0	-6.019058	-3.195034	-2.858383
29	6	0	-4.927959	-2.337831	-2.962181
30	8	0	-4.392418	1.903802	2.346605
31	8	0	-5.064600	-2.519165	3.607394
32	8	0	1.144249	2.933678	0.575322

33	6	0	2.393531	2.943855	0.750912
34	6	0	2.976752	4.196659	1.299488
35	6	0	4.351246	4.320189	1.517110
36	6	0	4.872015	5.497268	2.036828
37	6	0	4.026872	6.560165	2.342674
38	6	0	2.656342	6.441714	2.127347
39	6	0	2.131998	5.266614	1.608094
40	7	0	3.249754	1.970567	0.446029
41	6	0	2.886644	0.715878	0.216370
42	16	0	1.373717	-0.028408	0.686379
43	7	0	3.816886	-0.066893	-0.360769
44	6	0	3.588172	-1.473682	-0.732150
45	6	0	4.983000	-1.938253	-1.255164
46	6	0	5.747461	-0.654804	-1.567111
47	6	0	5.187617	0.416264	-0.612792
48	6	0	4.871448	-2.846151	-2.463027
49	8	0	4.504772	-4.081467	-2.088377
50	6	0	4.198231	-5.001040	-3.151073
51	6	0	5.106325	1.742804	-1.388261
52	8	0	5.989854	2.651952	-0.956238
53	6	0	5.957962	3.919600	-1.640500
54	6	0	5.982888	0.540225	0.712130
55	6	0	6.021859	-0.736954	1.507798
56	6	0	7.121301	-1.593809	1.434950
57	6	0	7.122295	-2.813602	2.102828
58	6	0	6.018677	-3.194196	2.858907
59	6	0	4.927488	-2.337070	2.962396
60	8	0	4.392231	1.903980	-2.346599
61	8	0	5.064587	-2.518881	-3.607015
62	1	0	6.821822	-0.775178	-1.442381
63	1	0	5.551743	-0.372739	-2.599772
64	1	0	5.462319	-2.498914	-0.456696
65	1	0	3.348468	-2.037733	0.163433
66	1	0	4.981897	4.384423	-1.511346
67	1	0	6.731336	4.520813	-1.171720
68	1	0	6.159947	3.783330	-2.701758
69	1	0	3.887389	-5.917377	-2.658416
70	1	0	3.391186	-4.603803	-3.766071
71	1	0	5.075828	-5.172273	-3.773196
72	1	0	6.993038	0.863243	0.463869
73	1	0	5.520635	1.330208	1.299003
74	1	0	4.066351	-2.615783	3.556100
75	1	0	7.982156	-1.305370	0.842725
76	1	0	4.999456	3.495011	1.264047
78	1	0	5.938102	5.586138	2.204724
79	1	0	1.996950	7.266730	2.365676
80	1	0	4.434587	7.477573	2.748864
81	1	0	-6.821562	-0.775478	1.442967
82	1	0	-5.551281	-0.372808	2.600051
83	1	0	-5.462130	-2.499230	0.457192
84	1	0	-3.348305	-2.038096	-0.163215
85	1	0	-4.981769	4.384256	1.510664
86	1	0	-6.731195	4.520612	1.171009
87	1	0	-6.159831	3.783384	2.701179
88	1	0	-3.886752	-5.917518	2.658877
89	1	0	-3.389886	-4.604035	3.766311
90	1	0	-5.074718	-5.171943	3.773863
91	1	0	-6.993242	0.862571	-0.463574
92	1	0	-5.521006	1.329509	-1.299004
93	1	0	-4.066934	-2.616510	-3.556062
94	1	0	-7.982231	-1.306231	-0.841873
95	1	0	-4.999938	3.494354	-1.264444
96	1	0	-1.071413	5.156389	-1.435334
97	1	0	-5.938991	5.585455	-2.204767
98	1	0	-1.998186	7.266907	-2.365243
99	1	0	-4.435857	7.477324	-2.748454
100	6	0	4.934728	-1.115470	2.300362
101	1	0	6.013131	-4.147175	3.372653
102	1	0	7.982876	-3.467042	2.030496
103	1	0	4.082135	-0.455614	2.397307
104	6	0	-4.935151	-1.116187	-2.300227
105	1	0	-4.082647	-0.456255	-2.397420
106	1	0	-6.013570	-4.148042	-3.372076
107	1	0	-7.983045	-3.467981	-2.029509
108	6	0	2.445634	-1.628080	-1.713385
109	6	0	1.553856	-2.695339	-1.622507
110	6	0	2.220635	-0.678714	-2.709621

111	6	0	0.448167	-2.804679	-2.453426
112	6	0	1.126059	-0.762355	-3.555845
113	1	0	2.889472	0.168581	-2.789726
114	6	0	0.243387	-1.821462	-3.406928
115	1	0	-0.251369	-3.616449	-2.330016
116	1	0	0.948220	-0.003007	-4.303377
117	17	0	-1.188955	-1.911434	-4.429579
118	17	0	1.761387	-3.937639	-0.387961
119	6	0	-2.445286	-1.627967	1.713410
120	6	0	-1.553303	-2.695061	1.622548
121	6	0	-2.220416	-0.678480	2.709533
122	6	0	-0.447577	-2.804133	2.453458
123	6	0	-1.125795	-0.761850	3.555739
124	1	0	-2.889350	0.168750	2.789563
125	6	0	-0.242959	-1.820823	3.406906
126	1	0	0.252106	-3.615780	2.330069
127	1	0	-0.948101	-0.002420	4.303223
128	17	0	1.189367	-1.910529	4.429604
129	17	0	-1.760563	-3.937472	0.388072

NiL1-B

Center Number	Atomic Number	Atomic Type	Coordinates (Angstroms) X	Y	Z
1	6	0	2.867425	-4.709915	-2.169731
2	6	0	3.887837	-3.827399	-1.815132
3	6	0	4.360605	-2.933610	-2.777252
4	6	0	3.799956	-2.889356	-4.047388
5	6	0	2.769977	-3.759814	-4.382596
6	6	0	2.314909	-4.679390	-3.443183
7	6	0	4.475929	-3.825573	-0.429661
8	6	0	3.945769	-2.752162	0.560141
9	7	0	4.004931	-1.404451	-0.024231
10	6	0	5.158515	-0.600486	0.426968
11	6	0	5.951405	-1.597285	1.316362
12	6	0	4.919051	-2.628260	1.754951
13	6	0	3.127381	-0.831510	-0.871369
14	7	0	3.518605	0.305369	-1.414418
15	6	0	2.680933	1.258507	-1.812439
16	8	0	1.493840	1.404237	-1.406350
17	28	0	0.457013	0.177011	-0.396256
18	16	0	-0.832015	-1.212249	0.702174
19	6	0	-2.344622	-0.319917	0.604984
20	7	0	-3.464421	-0.993275	0.276993
21	6	0	-4.761006	-0.291510	0.241388
22	6	0	-5.778377	-1.446625	0.001880
23	6	0	-4.954901	-2.580998	-0.600236
24	6	0	-3.538722	-2.436483	-0.003116
25	6	0	6.632002	-0.910331	2.482016
26	8	0	6.278373	-0.968382	3.631887
27	6	0	2.553839	-3.037848	1.159759
28	8	0	2.029653	-2.307462	1.963910
29	6	0	-6.928723	-1.035636	-0.893373
30	8	0	-7.056808	-1.335802	-2.053756
31	6	0	-2.531169	-2.794601	-1.117840
32	8	0	-2.392413	-2.136553	-2.118098
33	6	0	-3.342727	-3.293031	1.275126
34	6	0	-4.301122	-2.950154	2.382827
35	6	0	-5.444645	-3.719838	2.601909
36	6	0	-6.371752	-3.362194	3.574646
37	6	0	-6.164044	-2.226149	4.349324
38	6	0	-5.014638	-1.465506	4.158606
39	6	0	-4.088094	-1.829088	3.189061
40	6	0	3.213275	2.283372	-2.741459
41	6	0	4.535420	2.229449	-3.188388
42	6	0	5.015105	3.196594	-4.060157
43	6	0	4.180075	4.224110	-4.490018
44	6	0	2.861821	4.282572	-4.045153
45	6	0	2.378506	3.317573	-3.173347
46	16	0	1.565925	-1.580760	-1.180264
47	8	0	-0.375135	1.702326	0.337395
48	6	0	-1.481631	1.825581	0.934477
49	7	0	-2.491237	0.955349	0.908703

50	8	0	7.683555	-0.195864	2.051013
51	6	0	8.329302	0.636901	3.031861
52	8	0	2.084717	-4.245942	0.830131
53	6	0	0.787761	-4.577904	1.364939
54	6	0	-1.693895	3.084637	1.687546
55	6	0	-2.935187	3.385364	2.252750
56	6	0	-3.106407	4.563245	2.966174
57	6	0	-2.039359	5.442791	3.128519
58	6	0	-0.798726	5.143146	2.571515
59	6	0	-0.625446	3.971718	1.849490
60	8	0	-7.795998	-0.265265	-0.218092
61	6	0	-8.854708	0.325209	-0.992825
62	8	0	-1.933991	-3.976076	-0.914864
63	6	0	-0.976896	-4.367419	-1.921496
64	1	0	-5.378522	-3.559487	-0.383973
65	1	0	-4.921089	-2.455690	-1.680615
66	1	0	-6.190155	-1.728758	0.967860
67	1	0	-4.932486	0.152832	1.217014
68	1	0	-0.142761	-3.668872	-1.933300
69	1	0	-0.639977	-5.357501	-1.628513
70	1	0	-1.446022	-4.391479	-2.903529
71	1	0	-9.419369	0.933570	-0.292827
72	1	0	-8.435748	0.943104	-1.786551
73	1	0	-9.483862	-0.448275	-1.431387
74	1	0	-3.457354	-4.337368	0.988512
75	1	0	-2.317352	-3.164565	1.615755
76	1	0	-4.836472	-0.588141	4.767633
77	1	0	-5.613803	-4.604537	1.998975
78	1	0	-3.755564	2.697764	2.115290
79	1	0	0.330213	3.728700	1.407642
80	1	0	-4.073498	4.796653	3.393559
81	1	0	0.035049	5.821166	2.699955
82	1	0	-2.174147	6.360279	3.688134
83	1	0	5.363581	-3.590095	2.005364
84	1	0	4.392618	-2.257053	2.630749
85	1	0	6.728717	-2.049422	0.699487
86	1	0	5.750494	-0.313561	-0.436814
87	1	0	0.036659	-3.915384	0.939339
88	1	0	0.606889	-5.606958	1.067752
89	1	0	0.787470	-4.481461	2.449324
90	1	0	9.114241	1.159724	2.494095
91	1	0	7.613520	1.344331	3.449105
92	1	0	8.745463	0.028127	3.833463
93	1	0	5.552438	-3.658889	-0.511990
94	1	0	4.340478	-4.798416	0.040972
95	1	0	1.523473	-5.371493	-3.704328
96	1	0	5.162679	-2.250586	-2.524919
97	1	0	5.174403	1.434390	-2.833668
98	1	0	1.359416	3.348032	-2.814092
99	1	0	6.042005	3.153678	-4.400689
100	1	0	2.212064	5.082179	-4.377687
101	1	0	4.556345	4.979450	-5.168892
102	1	0	-6.886879	-1.941368	5.103247
103	1	0	-7.255402	-3.969431	3.726652
104	1	0	-3.193880	-1.234078	3.055305
105	1	0	2.494977	-5.407558	-1.432101
106	1	0	2.328215	-3.725880	-5.370337
107	1	0	4.164632	-2.172424	-4.771842
108	6	0	4.683781	0.666175	1.115704
109	6	0	5.298743	1.899725	0.907172
110	6	0	3.547159	0.643931	1.926938
111	6	0	4.811583	3.071528	1.470764
112	6	0	3.034425	1.797564	2.499077
113	1	0	3.021231	-0.289136	2.079421
114	6	0	3.675716	3.003294	2.261562
115	1	0	5.296328	4.016035	1.274894
116	1	0	2.139411	1.759969	3.102937
117	17	0	6.710644	2.048957	-0.142165
118	17	0	3.032933	4.486898	2.959887
119	6	0	-4.796801	0.805439	-0.800624
120	6	0	-5.465841	2.008625	-0.580822
121	6	0	-4.163789	0.638532	-2.032648
122	6	0	-5.516928	3.011992	-1.538941
123	6	0	-4.196306	1.622321	-3.008482
124	1	0	-3.610339	-0.271919	-2.220682
125	6	0	-4.877586	2.802073	-2.750155
126	1	0	-6.032443	3.938499	-1.335695

127	1	0	-3.693922	1.475694	-3.953806
128	17	0	-4.929107	4.068119	-3.972978
129	17	0	-6.276078	2.335608	0.955963

PdL1-A

Center Number	Atomic Number	Atomic Type	Coordinates (Angstroms) X	Y	Z
1	6	0	-4.420273	6.331444	-2.619566
2	6	0	-5.197725	5.249264	-2.217780
3	6	0	-4.600349	4.141019	-1.632950
4	6	0	-3.216778	4.106357	-1.443482
5	6	0	-2.440561	5.196436	-1.847400
6	6	0	-3.040798	6.301913	-2.433000
7	6	0	-2.548240	2.927209	-0.826411
8	8	0	-1.306497	3.022675	-0.636745
9	46	0	-0.000070	1.524684	0.000199
10	16	0	-1.460280	-0.092451	-0.745804
11	6	0	-2.955756	0.674325	-0.222815
12	7	0	-3.341608	1.913032	-0.477045
13	7	0	-3.853530	-0.106218	0.407922
14	6	0	-3.593468	-1.501196	0.806383
15	6	0	-4.962944	-1.970446	1.389956
16	6	0	-5.736930	-0.690036	1.689380
17	6	0	-5.224477	0.360515	0.687338
18	6	0	-4.796470	-2.839810	2.619771
19	8	0	-4.418065	-4.078831	2.269625
20	6	0	-4.056307	-4.959289	3.347996
21	6	0	-5.143329	1.710664	1.420412
22	8	0	-6.053707	2.591998	0.986567
23	6	0	-6.020662	3.882576	1.626790
24	6	0	-6.060300	0.430123	-0.616809
25	6	0	-6.088281	-0.865970	-1.381821
26	6	0	-7.158362	-1.752901	-1.253665
27	6	0	-7.145200	-2.986056	-1.896418
28	6	0	-6.057063	-3.349929	-2.682635
29	6	0	-4.996475	-2.463398	-2.841469
30	8	0	-4.403614	1.911994	2.351069
31	8	0	-4.959830	-2.482224	3.759256
32	8	0	1.306398	3.022463	0.637579
33	6	0	2.548257	2.927123	0.826497
34	6	0	3.216988	4.106277	1.443353
35	6	0	4.600669	4.141133	1.631983
36	6	0	5.198222	5.249381	2.216629
37	6	0	4.420841	6.331364	2.619077
38	6	0	3.041256	6.301639	2.433348
39	6	0	2.440842	5.196169	1.847921
40	7	0	3.341580	1.913067	0.476588
41	6	0	2.955739	0.674304	0.222609
42	16	0	1.460313	-0.092512	0.745706
43	7	0	3.853484	-0.106322	-0.408090
44	6	0	3.593384	-1.501353	-0.806309
45	6	0	4.962852	-1.970752	-1.389819
46	6	0	5.736905	-0.690423	-1.689394
47	6	0	5.224445	0.360316	-0.687544
48	6	0	4.796319	-2.840241	-2.619543
49	8	0	4.417965	-4.079239	-2.269261
50	6	0	4.056075	-4.959769	-3.347532
51	6	0	5.143345	1.710327	-1.420876
52	8	0	6.053842	2.591672	-0.987273
53	6	0	6.020827	3.882140	-1.627720
54	6	0	6.060246	0.430135	0.616608
55	6	0	6.088199	-0.865833	1.381834
56	6	0	7.158270	-1.752802	1.253857
57	6	0	7.145061	-2.985855	1.896802
58	6	0	6.056888	-3.349589	2.683033
59	6	0	4.996309	-2.463016	2.841690
60	8	0	4.403554	1.911551	-2.351491
61	8	0	4.959554	-2.482743	-3.759073
62	1	0	6.812804	-0.829625	-1.603120
63	1	0	5.512636	-0.375404	-2.706624
64	1	0	5.459178	-2.562752	-0.625150
65	1	0	3.378695	-2.085314	0.082482

66	1	0	5.055339	4.355067	-1.455622
67	1	0	6.815191	4.456962	-1.161057
68	1	0	6.191163	3.778908	-2.698206
69	1	0	3.745281	-5.885071	-2.871887
70	1	0	3.235253	-4.528737	-3.920105
71	1	0	4.907273	-5.127626	-4.006139
72	1	0	7.070794	0.734405	0.347054
73	1	0	5.635707	1.215071	1.237104
74	1	0	4.148288	-2.728281	3.460086
75	1	0	8.006928	-1.477391	0.638292
76	1	0	5.197704	3.305023	1.302627
77	1	0	1.371376	5.156794	1.696217
78	1	0	6.271440	5.268978	2.359667
79	1	0	2.433925	7.141518	2.746338
80	1	0	4.888032	7.194648	3.076608
81	1	0	-6.812839	-0.829214	1.603193
82	1	0	-5.512597	-0.374870	2.706550
83	1	0	-5.459324	-2.562495	0.625355
84	1	0	-3.378779	-2.085310	-0.082307
85	1	0	-5.055062	4.355332	1.454853
86	1	0	-6.814817	4.457434	1.159818
87	1	0	-6.191291	3.779561	2.697251
88	1	0	-3.745549	-5.884655	2.872454
89	1	0	-3.235491	-4.528265	3.920584
90	1	0	-4.907558	-5.127025	4.006566
91	1	0	-7.070842	0.734449	-0.347295
92	1	0	-5.635761	1.214957	-1.237434
93	1	0	-4.148487	-2.728773	-3.459863
94	1	0	-8.006988	-1.477376	-0.638107
95	1	0	-5.197442	3.304752	-1.304092
96	1	0	-1.371180	5.157198	-1.695059
97	1	0	-6.270857	5.268705	-2.361482
98	1	0	-2.433414	7.141945	-2.745478
99	1	0	-4.887323	7.194731	-3.077236
100	6	0	5.017948	-1.228615	2.204209
101	1	0	6.040336	-4.312515	3.177617
102	1	0	7.982575	-3.662706	1.781731
103	1	0	4.190516	-0.545210	2.345670
104	6	0	-5.018065	-1.228899	-2.204178
105	1	0	-4.190631	-0.545526	-2.345779
106	1	0	-6.040545	-4.312928	-3.177079
107	1	0	-7.982721	-3.662873	-1.781211
108	6	0	2.417407	-1.619300	-1.751692
109	6	0	1.518913	-2.680933	-1.657441
110	6	0	2.173987	-0.648412	-2.722312
111	6	0	0.392938	-2.766936	-2.463141
112	6	0	1.058882	-0.709006	-3.543713
113	1	0	2.846863	0.195704	-2.802412
114	6	0	0.173880	-1.766147	-3.395459
115	1	0	-0.310129	-3.575330	-2.337089
116	1	0	0.867967	0.066229	-4.271347
117	17	0	1.746151	-3.946666	-0.450568
118	17	0	-1.281145	-1.830770	-4.387108
119	6	0	-2.417480	-1.619015	1.751783
120	6	0	-1.518967	-2.680641	1.657631
121	6	0	-2.174047	-0.648010	2.722281
122	6	0	-0.392957	-2.766521	2.463299
123	6	0	-1.058899	-0.708471	3.543637
124	1	0	-2.846926	0.196111	2.802304
125	6	0	-0.173876	-1.765609	3.395477
126	1	0	0.310104	-3.574938	2.337339
127	1	0	-0.867965	0.066870	4.271150
128	17	0	1.281213	-1.830058	4.387042
129	17	0	-1.746193	-3.946534	0.450923

PdL1-B

Center Number	Atomic Number	Atomic Type	Coordinates (Angstroms) X	Y	Z
1	6	0	4.837504	-2.071845	-2.978506
2	6	0	4.803576	-3.197936	-2.152168
3	6	0	5.889531	-4.074441	-2.177867
4	6	0	6.997331	-3.817463	-2.978063

5	6	0	7.032450	-2.676324	-3.772048
6	6	0	5.944753	-1.808688	-3.775664
7	6	0	3.644788	-3.437018	-1.222367
8	6	0	3.708292	-2.594605	0.078460
9	7	0	3.827045	-1.155799	-0.205137
10	6	0	5.164430	-0.587426	0.046813
11	6	0	6.005545	-1.830701	0.461840
12	6	0	4.983161	-2.872936	0.904362
13	6	0	2.864659	-0.377962	-0.736280
14	7	0	3.211888	0.847215	-1.059042
15	6	0	2.385563	1.869636	-1.295107
16	8	0	1.212367	2.007865	-0.854567
17	46	0	0.015825	0.520229	-0.006345
18	16	0	-1.268236	-1.103660	0.994672
19	6	0	-2.849108	-0.353303	0.740318
20	7	0	-3.838980	-1.123549	0.246950
21	6	0	-5.180956	-0.545497	0.055548
22	6	0	-6.054692	-1.785011	-0.299080
23	6	0	-5.055165	-2.816128	-0.829011
24	6	0	-3.742646	-2.560302	-0.053007
25	6	0	7.010954	-1.515600	1.550175
26	8	0	6.896919	-1.803575	2.715016
27	6	0	2.503021	-2.846538	1.008932
28	8	0	2.249705	-2.159285	1.966170
29	6	0	-7.196211	-1.451739	-1.237829
30	8	0	-8.312081	-1.185328	-0.869417
31	6	0	-2.577176	-2.808455	-1.034852
32	8	0	-2.361287	-2.113795	-1.996353
33	6	0	-3.628312	-3.421561	1.232163
34	6	0	-4.736501	-3.188744	2.222735
35	6	0	-5.821697	-4.063271	2.295915
36	6	0	-6.886463	-3.810950	3.153652
37	6	0	-6.877242	-2.677435	3.958937
38	6	0	-5.787871	-1.813024	3.916041
39	6	0	-4.723619	-2.071038	3.060788
40	6	0	2.938663	2.991318	-2.096885
41	6	0	4.253770	2.960479	-2.565820
42	6	0	4.749452	4.015284	-3.319083
43	6	0	3.936425	5.106355	-3.611582
44	6	0	2.623921	5.140720	-3.147119
45	6	0	2.125355	4.089384	-2.391801
46	16	0	1.279769	-1.107027	-1.036424
47	8	0	-1.160013	2.014020	0.852277
48	6	0	-2.339509	1.894874	1.282224
49	7	0	-3.175797	0.878036	1.060411
50	8	0	8.056481	-0.845227	1.041766
51	6	0	9.009147	-0.340538	1.994703
52	8	0	1.868298	-3.990066	0.720751
53	6	0	0.752133	-4.316116	1.574374
54	6	0	-2.891668	3.038391	2.053168
55	6	0	-4.237485	3.071067	2.425992
56	6	0	-4.735126	4.149987	3.142933
57	6	0	-3.893246	5.200013	3.497716
58	6	0	-2.550361	5.170472	3.129751
59	6	0	-2.050190	4.096916	2.407367
60	8	0	-6.819241	-1.458769	-2.529274
61	6	0	-7.794004	-0.979097	-3.471958
62	8	0	-1.929847	-3.952459	-0.780116
63	6	0	-0.836907	-4.262646	-1.668893
64	1	0	-5.403796	-3.837746	-0.690446
65	1	0	-4.887539	-2.645809	-1.889467
66	1	0	-6.517192	-2.134560	0.618624
67	1	0	-5.509427	-0.135194	1.005886
68	1	0	-0.037151	-3.536967	-1.531210
69	1	0	-0.504012	-5.255766	-1.382478
70	1	0	-1.172611	-4.247522	-2.704162
71	1	0	-7.317292	-1.053723	-4.444807
72	1	0	-8.696433	-1.587612	-3.433084
73	1	0	-8.046782	0.056896	-3.248119
74	1	0	-3.613407	-4.465809	0.924149
75	1	0	-2.664761	-3.213255	1.693831
76	1	0	-5.764530	-0.936294	4.551013
77	1	0	-5.837058	-4.945990	1.667032
78	1	0	-4.883776	2.257061	2.135753
79	1	0	-1.012105	4.061051	2.108117
80	1	0	-5.781587	4.173928	3.419550
81	1	0	-1.894594	5.986843	3.404784

82	1	0	-4.282641	6.041281	4.057729
83	1	0	5.337693	-3.890429	0.754111
84	1	0	4.779761	-2.736462	1.964678
85	1	0	6.554416	-2.164762	-0.415295
86	1	0	5.542594	-0.184313	-0.887553
87	1	0	-0.048671	-3.595138	1.420395
88	1	0	0.435361	-5.309180	1.269845
89	1	0	1.057763	-4.308003	2.618990
90	1	0	9.747658	0.196417	1.407201
91	1	0	8.514389	0.331349	2.695455
92	1	0	9.470185	-1.160184	2.544236
93	1	0	3.606038	-4.485454	-0.931551
94	1	0	2.706640	-3.212083	-1.727032
95	1	0	5.955564	-0.926137	-4.402934
96	1	0	5.869676	-4.964109	-1.559114
97	1	0	4.878110	2.115306	-2.320167
98	1	0	1.110184	4.100885	-2.020726
99	1	0	5.772458	3.989418	-3.672711
100	1	0	1.990586	5.989113	-3.373454
101	1	0	4.324643	5.929555	-4.198617
102	1	0	-7.708722	-2.473509	4.621360
103	1	0	-7.723789	-4.496441	3.190223
104	1	0	-3.878644	-1.394897	3.042746
105	1	0	3.995036	-1.392609	-2.996701
106	1	0	7.895924	-2.470211	-4.391624
107	1	0	7.832092	-4.507228	-2.980853
108	6	0	-5.190820	0.564394	-0.972178
109	6	0	-6.080412	1.632339	-0.874213
110	6	0	-4.323500	0.545544	-2.064130
111	6	0	-6.126508	2.645194	-1.823837
112	6	0	-4.343636	1.542908	-3.025901
113	1	0	-3.609602	-0.262303	-2.155279
114	6	0	-5.251882	2.583878	-2.896124
115	1	0	-6.821624	3.464393	-1.717814
116	1	0	-3.658323	1.515688	-3.860986
117	17	0	-7.199288	1.753530	0.485802
118	17	0	-5.295286	3.860636	-4.108624
119	6	0	5.124971	0.525672	1.071271
120	6	0	5.934183	1.655770	0.963674
121	6	0	4.268365	0.450119	2.170019
122	6	0	5.908257	2.674865	1.906132
123	6	0	4.219125	1.452336	3.126219
124	1	0	3.605243	-0.399748	2.263485
125	6	0	5.045289	2.556854	2.983937
126	1	0	6.537832	3.544280	1.790945
127	1	0	3.543305	1.378026	3.965980
128	17	0	4.997194	3.844927	4.183123
129	17	0	7.033303	1.864876	-0.403891

L3-A

Center Number	Atomic Number	Atomic Type	Coordinates (Angstroms) X	Y	Z
1	6	0	7.185211	0.174441	0.201717
2	6	0	6.296209	1.030672	-0.440863
3	6	0	5.041641	0.572414	-0.823743
4	6	0	4.672456	-0.751357	-0.571403
5	6	0	5.576583	-1.611922	0.055457
6	6	0	6.824649	-1.148364	0.446667
7	6	0	3.330160	-1.299112	-0.929926
8	8	0	3.135134	-2.480863	-1.139272
9	7	0	2.320372	-0.348248	-0.925844
10	6	0	0.970670	-0.576408	-1.234770
11	16	0	0.512539	-1.355556	-2.641913
12	7	0	0.102001	-0.091836	-0.328843
13	6	0	-1.352644	-0.260874	-0.509282
14	6	0	-1.948177	0.666570	0.587550
15	6	0	-0.861214	0.739918	1.651207
16	6	0	0.462341	0.724789	0.860294
17	6	0	-3.274838	0.172872	1.115860
18	8	0	-4.246668	0.398926	0.222378
19	6	0	-5.555940	-0.115921	0.547800
20	6	0	1.519248	-0.024671	1.700877

21	8	0	2.536981	0.758824	2.050059
22	6	0	3.568386	0.162990	2.874436
23	6	0	0.918930	2.159467	0.466125
24	6	0	-0.013283	2.883617	-0.450552
25	6	0	-1.016701	3.844428	-0.071063
26	6	0	-1.706414	4.217567	-1.256784
27	6	0	-2.764397	5.125991	-1.246511
28	6	0	-3.134742	5.662976	-0.023556
29	6	0	-2.467533	5.306348	1.163336
30	6	0	-1.415910	4.405069	1.150373
31	8	0	1.410083	-1.182537	2.026450
32	8	0	-3.452689	-0.357758	2.187395
33	1	0	-0.941351	1.626418	2.274925
34	1	0	-0.920120	-0.137136	2.292418
35	1	0	-2.118285	1.643775	0.145493
36	1	0	-1.621402	0.104198	-1.498732
37	1	0	4.031295	-0.667433	2.346408
38	1	0	4.288460	0.955947	3.045110
39	1	0	3.139433	-0.181744	3.812894
40	1	0	-6.191187	0.162057	-0.287010
41	1	0	-5.512611	-1.199118	0.651778
42	1	0	-5.918751	0.330694	1.471676
43	1	0	1.041228	2.717395	1.393566
44	1	0	1.915309	2.134214	0.025562
45	1	0	0.446492	2.097130	-2.479154
46	1	0	-2.784264	5.746536	2.100357
47	1	0	-3.953627	6.369731	0.020086
48	1	0	-3.278579	5.398503	-2.159469
49	1	0	-0.910870	4.142204	2.071720
50	1	0	4.373533	1.244268	-1.347771
51	1	0	5.283594	-2.636156	0.241608
52	1	0	6.579958	2.054125	-0.647984
53	1	0	7.515491	-1.816201	0.944546
54	1	0	8.159049	0.535719	0.506275
55	1	0	2.569319	0.560930	-0.569030
56	6	0	-1.804450	-1.701828	-0.382930
57	6	0	-2.905067	-2.126461	-1.124567
58	6	0	-1.204276	-2.587899	0.507662
59	6	0	-3.413048	-3.410563	-0.978590
60	1	0	-3.372633	-1.450448	-1.828767
61	6	0	-1.695399	-3.879560	0.659816
62	1	0	-0.338242	-2.282127	1.078344
63	6	0	-2.798787	-4.273094	-0.081931
64	1	0	-4.265993	-3.735773	-1.557563
65	1	0	-1.224831	-4.567938	1.347973
66	17	0	-3.432874	-5.913247	0.111564
67	6	0	-0.118869	2.724774	-1.809909
68	7	0	-1.134216	3.518012	-2.295378
69	1	0	-1.400777	3.584880	-3.263648

L3-B

Center Number	Atomic Number	Atomic Type	Coordinates (Angstroms) X	Y	Z
1	6	0	-4.860090	0.114052	-1.113663
2	6	0	-3.603441	2.065361	-1.838572
3	6	0	-4.244858	2.075025	-3.076758
4	6	0	-5.191866	1.091293	-3.315788
5	6	0	-5.497659	0.120813	-2.343354
6	6	0	-3.039057	1.370880	0.284481
7	6	0	-3.893954	1.093402	-0.841502
8	6	0	-2.928202	0.597319	1.558950
9	6	0	-1.814757	-0.485753	1.556754
10	7	0	-0.542470	0.046193	1.025350
11	6	0	-0.228199	-0.428171	-0.341342
12	6	0	-1.484513	-1.258731	-0.729267
13	6	0	-2.107836	-1.660196	0.597780
14	6	0	0.208550	0.957459	1.678409
15	16	0	-0.112043	1.480126	3.241893
16	6	0	-1.142284	-2.430540	-1.621586
17	8	0	-1.104710	-3.588511	-1.277654
18	6	0	-1.579475	-1.102205	2.948042
19	8	0	-0.636655	-1.816190	3.206363

20	7	0	1.245407	1.471017	0.914123
21	6	0	2.486765	1.958634	1.318947
22	6	0	3.282004	2.529013	0.189992
23	6	0	2.682353	3.126879	-0.922670
24	6	0	3.469739	3.648728	-1.942105
25	6	0	4.856720	3.568433	-1.862258
26	6	0	5.458002	2.977541	-0.752953
27	6	0	4.674955	2.467100	0.273024
28	8	0	2.908071	1.892805	2.454974
29	8	0	-0.864492	-2.008276	-2.861438
30	6	0	-0.411969	-3.010563	-3.798171
31	8	0	-2.601555	-0.887650	3.774535
32	6	0	-2.490924	-1.469453	5.090829
33	1	0	-3.174884	-1.849632	0.517571
34	1	0	-1.626766	-2.566245	0.961209
35	1	0	-2.152617	-0.606924	-1.285174
36	1	0	-0.185946	0.422851	-1.023868
37	1	0	-1.625321	-1.054601	5.605226
38	1	0	-3.407807	-1.198080	5.604146
39	1	0	-2.394707	-2.551409	5.019612
40	1	0	-0.237903	-2.478909	-4.728073
41	1	0	0.509945	-3.464706	-3.437898
42	1	0	-1.174999	-3.775478	-3.929901
43	1	0	-3.868536	0.096076	1.785660
44	1	0	-2.724763	1.276091	2.382999
45	1	0	-1.556693	3.007144	0.517919
46	1	0	-6.244858	-0.631127	-2.563691
47	1	0	-5.706650	1.069554	-4.267938
48	1	0	-4.009735	2.822137	-3.824306
49	1	0	-5.107935	-0.637783	-0.374640
50	1	0	1.605562	3.222023	-0.985917
51	1	0	5.130593	2.008813	1.140450
52	1	0	3.000118	4.121986	-2.794676
53	1	0	6.536745	2.915241	-0.689661
54	1	0	5.468415	3.969971	-2.660216
55	1	0	1.161362	1.364489	-0.083777
56	6	0	1.083328	-1.183004	-0.410002
57	6	0	1.882321	-1.053709	-1.544017
58	6	0	1.494044	-2.022013	0.624149
59	6	0	3.075039	-1.757044	-1.658916
60	1	0	1.576419	-0.397934	-2.349590
61	6	0	2.685193	-2.729721	0.523944
62	1	0	0.893587	-2.111321	1.520091
63	6	0	3.459852	-2.590269	-0.619198
64	1	0	3.695158	-1.651551	-2.537824
65	1	0	3.007165	-3.378456	1.326532
66	17	0	4.977911	-3.485386	-0.748322
67	1	0	-2.236167	3.692917	-1.793702
68	7	0	-2.627758	2.891165	-1.327992
69	6	0	-2.298466	2.475834	-0.055510

NiL3-A

Center Number	Atomic Number	Atomic Type	Coordinates (Angstroms) X	Y	Z
1	6	0	3.095540	-0.275764	2.483599
2	6	0	4.454382	-0.128204	2.358897
3	6	0	5.051805	-1.176037	3.145898
4	6	0	3.991522	-1.933685	3.711775
5	7	0	2.813206	-1.361038	3.287259
6	6	0	4.222586	-3.045676	4.519846
7	6	0	5.539886	-3.400240	4.760226
8	6	0	6.605497	-2.665338	4.210811
9	6	0	6.373059	-1.561227	3.409137
10	6	0	5.172311	0.871337	1.510579
11	6	0	5.276442	0.468807	0.013054
12	6	0	6.098478	-0.820126	-0.205905
13	6	0	5.078529	-1.949614	-0.147061
14	6	0	3.789547	-1.344651	-0.773813
15	7	0	3.958530	0.096539	-0.536673
16	6	0	5.540903	-3.205115	-0.846916
17	8	0	4.716791	-4.232765	-0.562162
18	6	0	4.988114	-5.461376	-1.259680

19	6	0	3.587543	-1.699494	-2.232639
20	6	0	2.777661	-2.787330	-2.551907
21	6	0	2.609901	-3.191404	-3.868705
22	6	0	3.258825	-2.486183	-4.872754
23	6	0	4.058025	-1.391733	-4.581112
24	6	0	4.221189	-1.001916	-3.257045
25	6	0	5.897555	1.568885	-0.857882
26	8	0	6.652083	2.416831	-0.140634
27	6	0	7.354087	3.417994	-0.903983
28	6	0	2.950960	0.985689	-0.639090
29	16	0	1.414953	0.339962	-1.156212
30	28	0	-0.086636	1.819938	-0.577600
31	8	0	1.153029	3.225151	-0.506400
32	6	0	2.408480	3.262311	-0.379930
33	6	0	2.993711	4.622375	-0.220184
34	6	0	4.295204	4.800151	0.251986
35	6	0	4.809810	6.079815	0.412781
36	6	0	4.036375	7.190714	0.090271
37	6	0	2.738345	7.017787	-0.383082
38	6	0	2.214931	5.741149	-0.529045
39	17	0	3.047710	-2.986926	-6.552096
40	8	0	5.816218	1.609399	-2.059191
41	8	0	6.503096	-3.298678	-1.565318
42	7	0	3.267166	2.245587	-0.351330
43	8	0	-1.364167	3.139401	-0.187903
44	6	0	-2.610100	3.137809	0.005043
45	6	0	-3.248818	4.481014	0.087607
46	6	0	-4.637311	4.626775	0.107777
47	6	0	-5.205383	5.892332	0.166924
48	6	0	-4.393582	7.021567	0.217245
49	6	0	-3.008337	6.881075	0.200756
50	6	0	-2.437289	5.618597	0.130191
51	7	0	-3.417290	2.090095	0.170395
52	6	0	-3.031068	0.822425	0.065322
53	16	0	-1.524930	0.180621	-0.527925
54	7	0	-3.953500	-0.093953	0.429008
55	6	0	-3.697414	-1.541165	0.426878
56	6	0	-5.120150	-2.142658	0.610194
57	6	0	-5.916105	-1.045791	1.306061
58	6	0	-5.345287	0.276732	0.751505
59	6	0	-5.108221	-3.447423	1.370863
60	8	0	-4.588569	-4.429566	0.608530
61	6	0	-4.418386	-5.701978	1.258831
62	6	0	-2.697215	-1.966640	1.484888
63	6	0	-1.827044	-3.018821	1.209364
64	6	0	-0.918680	-3.463740	2.160256
65	6	0	-0.891864	-2.835616	3.396514
66	6	0	-1.741509	-1.782713	3.694930
67	6	0	-2.646334	-1.351388	2.732520
68	6	0	-5.345561	1.306084	1.892416
69	8	0	-6.299631	2.235133	1.742358
70	6	0	-6.389502	3.208911	2.801532
71	6	0	-6.092562	0.784789	-0.510432
72	6	0	-5.999589	-0.135091	-1.684260
73	6	0	-6.948763	-1.145283	-2.075973
74	6	0	-6.407130	-1.818307	-3.203683
75	7	0	-5.188656	-1.233728	-3.474986
76	6	0	-4.954078	-0.219975	-2.566310
77	6	0	-7.069743	-2.872550	-3.828803
78	6	0	-8.295398	-3.256942	-3.309849
79	6	0	-8.850828	-2.606282	-2.194303
80	6	0	-8.190826	-1.558109	-1.576558
81	17	0	0.268056	-3.383025	4.619413
82	8	0	-4.641579	1.227767	2.868233
83	8	0	-5.494462	-3.609247	2.499997
84	1	0	-6.984119	-1.128125	1.119701
85	1	0	-5.751048	-1.109803	2.380253
86	1	0	-5.526826	-2.341098	-0.378474
87	1	0	-3.311081	-1.833034	-0.548677
88	1	0	-5.459777	3.770949	2.868999
89	1	0	-7.211561	3.861933	2.523676
90	1	0	-6.588726	2.716264	3.751904
91	1	0	-4.010865	-6.363990	0.500166
92	1	0	-3.727215	-5.603330	2.095609
93	1	0	-5.374565	-6.075560	1.622234
94	1	0	-7.134602	0.948244	-0.237834
95	1	0	-5.674867	1.754030	-0.768377

96	1	0	-4.044075	0.355907	-2.612370
97	1	0	-9.812032	-2.931538	-1.817347
98	1	0	-8.835790	-4.073199	-3.771788
99	1	0	-6.643968	-3.375413	-4.688385
100	1	0	-8.633880	-1.061006	-0.722307
101	1	0	-5.260345	3.746407	0.081116
102	1	0	-1.364068	5.490527	0.111904
103	1	0	-6.283110	5.998193	0.174244
104	1	0	-2.374214	7.757698	0.241789
105	1	0	-4.838226	8.007759	0.267582
106	1	0	6.878386	-0.926265	0.543976
107	1	0	6.567193	-0.812606	-1.188811
108	1	0	4.857017	-2.210395	0.884712
109	1	0	2.918628	-1.684796	-0.214060
110	1	0	6.644730	4.047606	-1.438159
111	1	0	7.910731	3.998538	-0.173904
112	1	0	8.027763	2.944916	-1.616900
113	1	0	4.247096	-6.169676	-0.900058
114	1	0	4.887020	-5.310105	-2.334208
115	1	0	5.994829	-5.812196	-1.037250
116	1	0	6.185221	1.021827	1.882131
117	1	0	4.669143	1.832906	1.558479
118	1	0	2.295670	0.300439	2.047746
119	1	0	7.622549	-2.970146	4.422397
120	1	0	5.753612	-4.259692	5.382841
121	1	0	3.402591	-3.613209	4.939938
122	1	0	7.203318	-1.000291	2.997436
123	1	0	4.887993	3.933683	0.500205
124	1	0	1.205942	5.589442	-0.886003
125	1	0	5.815924	6.211249	0.791255
126	1	0	2.134217	7.880200	-0.635358
127	1	0	4.441620	8.187771	0.210372
128	1	0	2.266269	-3.321956	-1.761469
129	1	0	4.826189	-0.134791	-3.029004
130	1	0	1.977458	-4.032714	-4.116101
131	1	0	4.546759	-0.847891	-5.377357
132	1	0	-1.848016	-3.488249	0.234153
133	1	0	-3.293320	-0.512776	2.951738
134	1	0	-0.235840	-4.273213	1.942802
135	1	0	-1.700030	-1.304317	4.663536
136	1	0	-4.583733	-1.472304	-4.241030
137	1	0	1.893991	-1.670345	3.557648

NiL3-A

Center Number	Atomic Number	Atomic Type	Coordinates (Angstroms) X	Y	Z
1	6	0	-2.065205	-6.882702	-2.872303
2	6	0	-3.147902	-6.036844	-2.648557
3	6	0	-2.952943	-4.799467	-2.051266
4	6	0	-1.670420	-4.401966	-1.666423
5	6	0	-0.587106	-5.257843	-1.883427
6	6	0	-0.785217	-6.490445	-2.489142
7	6	0	-1.442412	-3.085569	-1.015981
8	8	0	-0.304084	-2.909147	-0.496772
9	28	0	0.551751	-1.390110	0.211915
10	16	0	-0.983974	-0.004021	-0.475542
11	6	0	-2.424150	-0.991867	-0.617102
12	7	0	-2.484235	-2.253228	-1.000054
13	7	0	-3.603168	-0.375749	-0.398029
14	6	0	-4.878374	-1.033150	-0.736664
15	6	0	-5.883448	0.144242	-0.587919
16	6	0	-5.255623	1.020441	0.484711
17	6	0	-3.753307	0.998631	0.130142
18	6	0	-7.283485	-0.307969	-0.251271
19	8	0	-7.868321	-0.879656	-1.324885
20	6	0	-9.166845	-1.455951	-1.091302
21	6	0	-2.966953	1.098961	1.455438
22	8	0	-2.410765	2.301364	1.647327
23	6	0	-1.673410	2.454456	2.879338
24	6	0	-3.338873	2.065024	-0.947184
25	6	0	-4.352394	3.099995	-1.317174
26	6	0	-5.013051	3.167843	-2.515564

27	6	0	-5.736062	4.942855	-1.338926
28	6	0	-4.510818	4.706975	0.743893
29	6	0	-5.132512	5.860240	1.190072
30	6	0	-6.049223	6.552439	0.380391
31	6	0	-6.365523	6.102411	-0.891008
32	8	0	-2.946876	0.219555	2.279334
33	8	0	-7.820516	-0.190879	0.819160
34	8	0	1.890164	-2.568454	0.835121
35	6	0	3.049397	-2.360469	1.292897
36	6	0	3.737931	-3.524822	1.906193
37	6	0	5.086792	-3.455612	2.262410
38	6	0	5.714695	-4.555999	2.828559
39	6	0	5.001191	-5.731171	3.046855
40	6	0	3.656670	-5.804734	2.692268
41	6	0	3.026708	-4.708630	2.120452
42	7	0	3.751877	-1.229225	1.226722
43	6	0	3.241863	-0.056585	0.908823
44	16	0	1.545002	0.385838	1.009551
45	7	0	4.119095	0.905257	0.555939
46	6	0	5.572808	0.673974	0.658329
47	6	0	6.138861	2.094235	0.364556
48	6	0	5.102643	2.713931	-0.563324
49	6	0	3.756709	2.235323	0.014750
50	6	0	7.531578	2.067193	-0.220135
51	8	0	8.431018	1.708027	0.717654
52	6	0	9.780405	1.524044	0.249376
53	6	0	2.781138	2.032361	-1.164551
54	8	0	1.839295	2.963625	-1.219648
55	6	0	0.883532	2.853913	-2.291778
56	6	0	3.272421	3.149921	1.177495
57	6	0	2.927418	4.576655	0.885553
58	6	0	1.597946	5.111238	0.728714
59	6	0	1.721507	6.509940	0.540518
60	6	0	0.613571	7.335153	0.345812
61	6	0	-0.636581	6.738324	0.343985
62	6	0	-0.781604	5.351503	0.529996
63	6	0	0.320402	4.537461	0.721694
64	8	0	2.931862	1.170927	-1.997272
65	8	0	7.818074	2.323379	-1.360656
66	1	0	5.152305	3.798545	-0.609361
67	1	0	5.245683	2.325288	-1.569643
68	1	0	6.190239	2.636034	1.310408
69	1	0	5.799222	0.382385	1.683080
70	1	0	0.346419	1.910304	-2.209044
71	1	0	0.211325	3.695530	-2.154706
72	1	0	1.389210	2.907551	-3.254741
73	1	0	10.358344	1.251611	1.127787
74	1	0	9.809812	0.727930	-0.493994
75	1	0	10.160111	2.444448	-0.191831
76	1	0	2.411209	2.671820	1.637044
77	1	0	4.068340	3.115556	1.925070
78	1	0	4.855950	5.679073	0.906662
79	1	0	-1.772878	4.920364	0.511184
80	1	0	-1.520065	7.345694	0.193872
81	1	0	0.724241	8.402772	0.199377
82	1	0	0.202757	3.468540	0.835049
83	1	0	5.628297	-2.541220	2.072068
84	1	0	1.985677	-4.749917	1.831866
85	1	0	6.762585	-4.500631	3.095886
86	1	0	3.100887	-6.718628	2.859983
87	1	0	5.492433	-6.589136	3.489070
88	1	0	-5.658276	2.027394	0.499565
89	1	0	-5.417295	0.565622	1.460273
90	1	0	-5.922602	0.680936	-1.536580
91	1	0	-4.828080	-1.360746	-1.774324
92	1	0	-0.828802	1.766547	2.885411
93	1	0	-1.330411	3.483991	2.883025
94	1	0	-2.317974	2.248507	3.732026
95	1	0	-9.482330	-1.860574	-2.048821
96	1	0	-9.094892	-2.245365	-0.343623
97	1	0	-9.865225	-0.695140	-0.745470
98	1	0	-2.427980	2.552199	-0.608458
99	1	0	-3.068070	1.516181	-1.847991
100	1	0	-4.938336	2.519272	-3.373690
101	1	0	-6.520445	7.450635	0.758659
102	1	0	-4.912691	6.237319	2.180733
103	1	0	-3.813962	4.174085	1.374498

104	1	0	-7.076668	6.633667	-1.511692
105	1	0	-3.783285	-4.138251	-1.854531
106	1	0	0.398026	-4.940932	-1.570521
107	1	0	-4.145475	-6.345675	-2.935120
108	1	0	0.057553	-7.148236	-2.660368
109	1	0	-2.218745	-7.847520	-3.339821
110	6	0	-5.208473	-2.240290	0.114945
111	6	0	-5.979390	-3.258174	-0.443678
112	6	0	-4.793688	-2.355427	1.438389
113	6	0	-6.345256	-4.371409	0.300894
114	1	0	-6.296775	-3.184376	-1.476575
115	6	0	-5.148636	-3.465955	2.194210
116	1	0	-4.168633	-1.590761	1.878929
117	6	0	-5.924049	-4.461727	1.619454
118	1	0	-6.936126	-5.163635	-0.137476
119	1	0	-4.819019	-3.558604	3.219629
120	17	0	-6.372834	-5.875580	2.574009
121	6	0	6.108554	-0.405461	-0.258887
122	6	0	7.210812	-1.147553	0.161189
123	6	0	5.559096	-0.661354	-1.511844
124	6	0	7.769673	-2.124225	-0.651789
125	1	0	7.637802	-0.965713	1.139780
126	6	0	6.105358	-1.638819	-2.334351
127	1	0	4.682749	-0.119823	-1.840910
128	6	0	7.207832	-2.358200	-1.898324
129	1	0	8.618543	-2.705329	-0.319234
130	1	0	5.672111	-1.843378	-3.303456
131	17	0	7.901676	-3.603049	-2.937879
132	1	0	3.466497	7.725309	0.503814
133	6	0	3.783268	5.643899	0.805590
134	6	0	-4.799677	4.231955	-0.542709
135	1	0	-6.413922	4.553580	-3.315340
136	7	0	-5.850484	4.266555	-2.534741
137	7	0	3.067918	6.807073	0.586422

PdL3-A

Center Number	Atomic Number	Atomic Type	Coordinates (Angstroms) X	Y	Z
1	6	0	-5.361494	-0.983803	2.013562
2	6	0	-4.491104	-2.119413	1.488784
3	6	0	-3.204801	-1.428668	0.945687
4	6	0	-5.040130	0.225408	1.112497
5	1	0	-6.420954	-1.229215	1.988983
6	1	0	-5.082748	-0.767125	3.043375
7	1	0	-4.974847	-2.617923	0.652979
8	1	0	-2.942140	-1.886492	-0.005119
9	7	0	-3.647789	-0.042777	0.711771
10	6	0	-5.069820	1.488898	1.986559
11	8	0	-4.299050	1.687952	2.892040
12	8	0	-6.104431	2.290672	1.699627
13	6	0	-6.188642	3.497717	2.482064
14	1	0	-5.302905	4.108703	2.315286
15	1	0	-7.078386	4.010149	2.128255
16	1	0	-6.272357	3.258600	3.541043
17	6	0	-4.187286	-3.169035	2.531304
18	8	0	-4.383960	-3.068002	3.715120
19	8	0	-3.643646	-4.258936	1.953249
20	6	0	-3.196991	-5.286753	2.855198
21	1	0	-2.811465	-6.081371	2.222684
22	1	0	-2.413469	-4.897709	3.505181
23	1	0	-4.025123	-5.647340	3.463611
24	6	0	-5.948825	0.331849	-0.145138
25	1	0	-6.985282	0.369288	0.190040
26	1	0	-5.735399	1.284292	-0.622117
27	6	0	-5.744734	-0.775743	-1.128984
28	6	0	-6.470627	-2.016119	-1.220684
29	1	0	-3.986349	-0.120276	-2.320571
30	6	0	-5.843810	-2.796874	-2.230611
31	6	0	-7.567846	-2.554409	-0.535529
32	6	0	-8.005676	-3.827481	-0.858178

33	6	0	-7.369177	-4.581925	-1.859325
34	1	0	-8.853430	-4.251708	-0.335384
35	6	0	-6.284276	-4.077570	-2.558147
36	1	0	-7.735072	-5.574362	-2.089495
37	1	0	-5.796779	-4.659492	-3.330646
38	1	0	-8.070275	-1.982356	0.235004
39	6	0	-2.897389	0.872408	0.072620
40	7	0	-3.384161	2.097269	-0.009682
41	6	0	-2.708571	3.178849	-0.418766
42	8	0	-1.459538	3.344927	-0.436173
43	6	0	-3.537668	4.341669	-0.842944
44	6	0	-4.933356	4.291357	-0.803898
45	6	0	-2.904944	5.502036	-1.298188
46	6	0	-5.683582	5.384677	-1.215044
47	1	0	-5.417526	3.398870	-0.438834
48	6	0	-3.657950	6.592440	-1.709516
49	1	0	-1.824700	5.528729	-1.323248
50	6	0	-5.048641	6.536982	-1.668806
51	1	0	-6.764977	5.337730	-1.182457
52	1	0	-3.161310	7.487355	-2.062664
53	1	0	-5.635039	7.388772	-1.990471
54	16	0	-1.436367	0.264795	-0.689288
55	6	0	5.361456	-0.983909	-2.013672
56	6	0	4.490993	-2.119508	-1.488987
57	6	0	3.204742	-1.428739	-0.945818
58	6	0	5.040157	0.225258	-1.112525
59	1	0	6.420900	-1.229386	-1.989086
60	1	0	5.082757	-0.767155	-3.043482
61	1	0	4.974730	-2.618130	-0.653241
62	1	0	2.942070	-1.886645	0.004946
63	7	0	3.647817	-0.042890	-0.711770
64	6	0	5.069873	1.488796	-1.986509
65	8	0	4.299084	1.687944	-2.891955
66	8	0	6.104521	2.290501	-1.699545
67	6	0	6.188800	3.497577	-2.481930
68	1	0	5.303098	4.108607	-2.315126
69	1	0	7.078571	4.009944	-2.128094
70	1	0	6.272506	3.258496	-3.540916
71	6	0	4.187135	-3.169033	-2.531593
72	8	0	4.383885	-3.067946	-3.715392
73	8	0	3.643274	-4.258881	-1.953647
74	6	0	3.196511	-5.286568	-2.855686
75	1	0	2.810826	-6.081168	-2.223246
76	1	0	2.413083	-4.897362	-3.505687
77	1	0	4.024619	-5.647245	-3.464078
78	6	0	5.948917	0.331610	0.145079
79	1	0	6.985363	0.368964	-0.190142
80	1	0	5.735582	1.284070	0.622064
81	6	0	5.744766	-0.775953	1.128947
82	6	0	6.470540	-2.016405	1.220664
83	1	0	3.986543	-0.120237	2.320637
84	6	0	5.843709	-2.797039	2.230677
85	6	0	7.567631	-2.554854	0.535432
86	6	0	8.005333	-3.827970	0.858093
87	6	0	7.368823	-4.582294	1.859320
88	1	0	8.852983	-4.252329	0.335239
89	6	0	6.284045	-4.077779	2.558217
90	1	0	7.734607	-5.574771	2.089497
91	1	0	5.796547	-4.659618	3.330779
92	1	0	8.070064	-1.982895	-0.235165
93	6	0	2.897493	0.872276	-0.072507
94	7	0	3.384317	2.097119	0.009863
95	6	0	2.708787	3.178680	0.419089
96	8	0	1.459759	3.344755	0.436745
97	6	0	3.537950	4.341526	0.843106
98	6	0	4.933633	4.291199	0.803945
99	6	0	2.905281	5.501938	1.298309
100	6	0	5.683906	5.384546	1.214935
101	1	0	5.417766	3.398677	0.438920
102	6	0	3.658333	6.592372	1.709477
103	1	0	1.825040	5.528645	1.323464
104	6	0	5.049019	6.536897	1.668653
105	1	0	6.765298	5.337581	1.182260
106	1	0	3.161731	7.487322	2.062592
107	1	0	5.635454	7.388711	1.990190
108	16	0	1.436453	0.264658	0.689349
109	46	0	0.000058	1.900201	0.000176

110	6	0	1.995997	-1.505682	-1.857995
111	6	0	1.052977	-2.507984	-1.642890
112	6	0	1.802807	-0.605431	-2.901989
113	6	0	-0.072185	-2.617803	-2.447109
114	1	0	1.187332	-3.197147	-0.819404
115	6	0	0.675924	-0.695902	-3.711022
116	1	0	2.515047	0.192201	-3.069381
117	6	0	-0.246969	-1.700690	-3.471702
118	1	0	-0.813349	-3.383515	-2.264873
119	1	0	0.514242	0.015405	-4.508399
120	17	0	-1.694510	-1.799792	-4.489684
121	6	0	-1.996073	-1.505767	1.857862
122	6	0	-1.053142	-2.508141	1.642687
123	6	0	-1.802803	-0.605605	2.901916
124	6	0	0.071976	-2.618156	2.446939
125	1	0	-1.187534	-3.197212	0.819129
126	6	0	-0.675961	-0.696270	3.710979
127	1	0	-2.514966	0.192088	3.069350
128	6	0	0.246816	-1.701152	3.471619
129	1	0	0.813070	-3.383925	2.264654
130	1	0	-0.514213	0.014971	4.508403
131	17	0	1.694298	-1.800501	4.489659
132	1	0	-4.139046	-2.337000	-3.431804
133	7	0	-4.800864	-2.048562	-2.730505
134	7	0	4.800881	-2.048586	2.730603
135	1	0	4.139062	-2.336914	3.431947
136	6	0	4.748680	-0.839509	2.070245
137	6	0	-4.748582	-0.839453	-2.070200

PdL3-B

Center Number	Atomic Number	Atomic Type	Coordinates (Angstroms) X	Y	Z
1	6	0	4.332467	5.625417	-3.000469
2	6	0	5.112324	4.543006	-2.602617
3	6	0	4.525059	3.460909	-1.962096
4	6	0	3.150574	3.454051	-1.712131
5	6	0	2.372670	4.544659	-2.108860
6	6	0	2.962509	5.623284	-2.752777
7	6	0	2.500904	2.302383	-1.029037
8	8	0	1.290569	2.446251	-0.704934
9	46	0	0.001777	0.970357	0.013729
10	16	0	1.336512	-0.677071	-0.875416
11	6	0	2.911671	0.029160	-0.496647
12	7	0	3.290097	1.259423	-0.774580
13	7	0	3.845923	-0.772552	0.042432
14	6	0	5.189939	-0.236056	0.325654
15	6	0	6.005705	-1.514991	0.663926
16	6	0	4.964177	-2.521090	1.137081
17	6	0	3.699639	-2.214541	0.303403
18	6	0	7.093409	-1.263008	1.681005
19	8	0	8.094032	-0.548109	1.130189
20	6	0	9.136791	-0.135791	2.032288
21	6	0	2.476073	-2.459122	1.209853
22	8	0	1.824206	-3.584341	0.887630
23	6	0	0.667533	-3.886000	1.694172
24	6	0	3.633143	-3.033576	-1.015081
25	6	0	4.694748	-2.689283	-2.008137
26	6	0	5.960385	-3.351064	-2.194873
27	6	0	6.664006	-2.638795	-3.202374
28	6	0	7.941386	-3.007838	-3.618505
29	6	0	8.520817	-4.109048	-3.009389
30	6	0	7.844124	-4.828599	-2.009190
31	6	0	6.574313	-4.460600	-1.599250
32	8	0	2.208965	-1.779490	2.168484
33	8	0	7.086196	-1.632617	2.827120
34	8	0	-1.254914	2.471640	0.731070
35	6	0	-2.468080	2.355214	1.055831
36	6	0	-3.093949	3.524692	1.729751
37	6	0	-4.474321	3.581505	1.937855
38	6	0	-5.039520	4.680682	2.569094
39	6	0	-4.231605	5.728853	3.001050
40	6	0	-2.855663	5.676716	2.795128

41	6	0	-2.287713	4.582166	2.158612
42	7	0	-3.279145	1.326756	0.809407
43	6	0	-2.928396	0.087027	0.539546
44	16	0	-1.364088	-0.650000	0.901042
45	7	0	-3.886868	-0.700462	0.019536
46	6	0	-5.222695	-0.140622	-0.248300
47	6	0	-6.072682	-1.408349	-0.552011
48	6	0	-5.053854	-2.425425	-1.060482
49	6	0	-3.770275	-2.144967	-0.242932
50	6	0	-7.234120	-1.081357	-1.461689
51	8	0	-6.967300	-1.312528	-2.756615
52	6	0	-7.956396	-0.849593	-3.694285
53	6	0	-2.563714	-2.411693	-1.166619
54	8	0	-1.923730	-3.545172	-0.850089
55	6	0	-0.777691	-3.862147	-1.665548
56	6	0	-3.697153	-2.966556	1.072822
57	6	0	-4.754350	-2.629268	2.072240
58	6	0	-6.002267	-3.317941	2.277469
59	6	0	-6.714324	-2.609837	3.281438
60	6	0	-7.979215	-3.003754	3.712453
61	6	0	-8.536286	-4.126355	3.122383
62	6	0	-7.850568	-4.842533	2.125932
63	6	0	-6.593707	-4.449168	1.700411
64	8	0	-2.299539	-1.739683	-2.131883
65	8	0	-8.274862	-0.617703	-1.063264
66	1	0	-5.389447	-3.452036	-0.931583
67	1	0	-4.858732	-2.252099	-2.116288
68	1	0	-6.510387	-1.746870	0.382307
69	1	0	-5.584019	0.329324	0.664649
70	1	0	0.000718	-3.117789	-1.505567
71	1	0	-0.444488	-4.839665	-1.330060
72	1	0	-1.053412	-3.884763	-2.718333
73	1	0	-7.576798	-1.123487	-4.674163
74	1	0	-8.917197	-1.325140	-3.502459
75	1	0	-8.062953	0.231527	-3.612825
76	1	0	-3.752348	-4.021301	0.805469
77	1	0	-2.712441	-2.812420	1.508442
78	1	0	-3.992998	-0.813296	3.094756
79	1	0	-8.316800	-5.715682	1.687702
80	1	0	-9.519188	-4.457069	3.432335
81	1	0	-8.511491	-2.452169	4.477542
82	1	0	-6.073988	-5.012957	0.935266
83	1	0	-5.087185	2.764547	1.586445
84	1	0	-1.222188	4.528758	1.984421
85	1	0	-6.110877	4.724030	2.720493
86	1	0	-2.226158	6.491716	3.129220
87	1	0	-4.673536	6.585808	3.494207
88	1	0	5.287672	-3.550859	1.005931
89	1	0	4.765757	-2.357627	2.194987
90	1	0	6.483299	-1.858175	-0.250646
91	1	0	5.572796	0.215320	-0.587836
92	1	0	-0.100521	-3.132635	1.526758
93	1	0	0.325536	-4.860219	1.357871
94	1	0	0.934560	-3.909979	2.749125
95	1	0	9.852198	0.406926	1.421025
96	1	0	8.724548	0.510042	2.807153
97	1	0	9.605716	-1.002753	2.495404
98	1	0	3.687642	-4.089094	-0.750327
99	1	0	2.651328	-2.878401	-1.456592
100	1	0	3.900039	-0.904950	-3.059261
101	1	0	8.326839	-5.685049	-1.556161
102	1	0	9.513035	-4.421283	-3.308814
103	1	0	8.465393	-2.454790	-4.388357
104	1	0	6.062710	-5.026961	-0.830784
105	1	0	5.115748	2.617123	-1.637128
106	1	0	1.311538	4.529371	-1.903269
107	1	0	6.179264	4.546729	-2.788097
108	1	0	2.354709	6.464826	-3.060173
109	1	0	4.791520	6.469633	-3.499919
110	6	0	5.182178	0.820557	1.409528
111	6	0	6.090961	1.873240	1.332392
112	6	0	4.310091	0.756524	2.493189
113	6	0	6.145220	2.846019	2.321020
114	1	0	6.764762	1.937955	0.487310
115	6	0	4.349715	1.725624	3.488227
116	1	0	3.576835	-0.036419	2.555280
117	6	0	5.270073	2.758396	3.394382

118	1	0	6.844913	3.667499	2.254579
119	1	0	3.667589	1.679443	4.325568
120	17	0	5.322894	3.993345	4.653520
121	6	0	-5.221022	0.898877	-1.348802
122	6	0	-6.189236	1.900038	-1.325184
123	6	0	-4.313565	0.857557	-2.404088
124	6	0	-6.267264	2.843143	-2.341657
125	1	0	-6.901023	1.941394	-0.510181
126	6	0	-4.375657	1.798317	-3.424351
127	1	0	-3.545124	0.096783	-2.430436
128	6	0	-5.355596	2.779296	-3.385182
129	1	0	-7.017956	3.620695	-2.319736
130	1	0	-3.665683	1.771075	-4.239228
131	17	0	-5.440758	3.974175	-4.681630
132	1	0	-6.147285	-0.880747	4.380707
133	6	0	-4.746819	-1.567687	2.938981
134	6	0	4.670127	-1.639414	-2.888584
135	1	0	6.062331	-0.941549	-4.332781
136	7	0	5.852516	-1.598257	-3.601756
137	7	0	-5.924054	-1.545891	3.661378

5. Reference

[1] S. Belveren, S. Poyraz, Christopher M. Pask, M. Ulger, H.A. Dondas, J.M. Sansano, Inorganica Chimica Acta 498 (2019) 119154.
